# Correction: *Nicotiana* Small RNA Sequences Support a Host Genome Origin of *Cucumber Mosaic Virus* Satellite RNA

**DOI:** 10.1371/journal.pgen.1005196

**Published:** 2015-04-27

**Authors:** The PLOS Genetics Staff

## Notice of Republication

This article was republished on April 2nd, 2015, to correct errors in the footer year, the citation year and copyright year that were introduced during the typesetting process. The publisher apologizes for the errors. Please download this article again to view the correct version. The originally published, uncorrected article and the republished, corrected article are provided here for reference.

## Supporting Information

S1 File Originally published, uncorrected article. (PDF)

S2 File Republished corrected article. (PDF)
