# Supplementary figures and images for: Nicotiana Small RNA Sequences Support a Host Genome Origin of Cucumber Mosaic Virus Satellite RNA
Source: PLoS Genet. 2015 Jan 8;11(1):e1004906. doi: 10.1371/journal.pgen.1004906 (PMC4287446; doi:10.1371/journal.pgen.1004906)

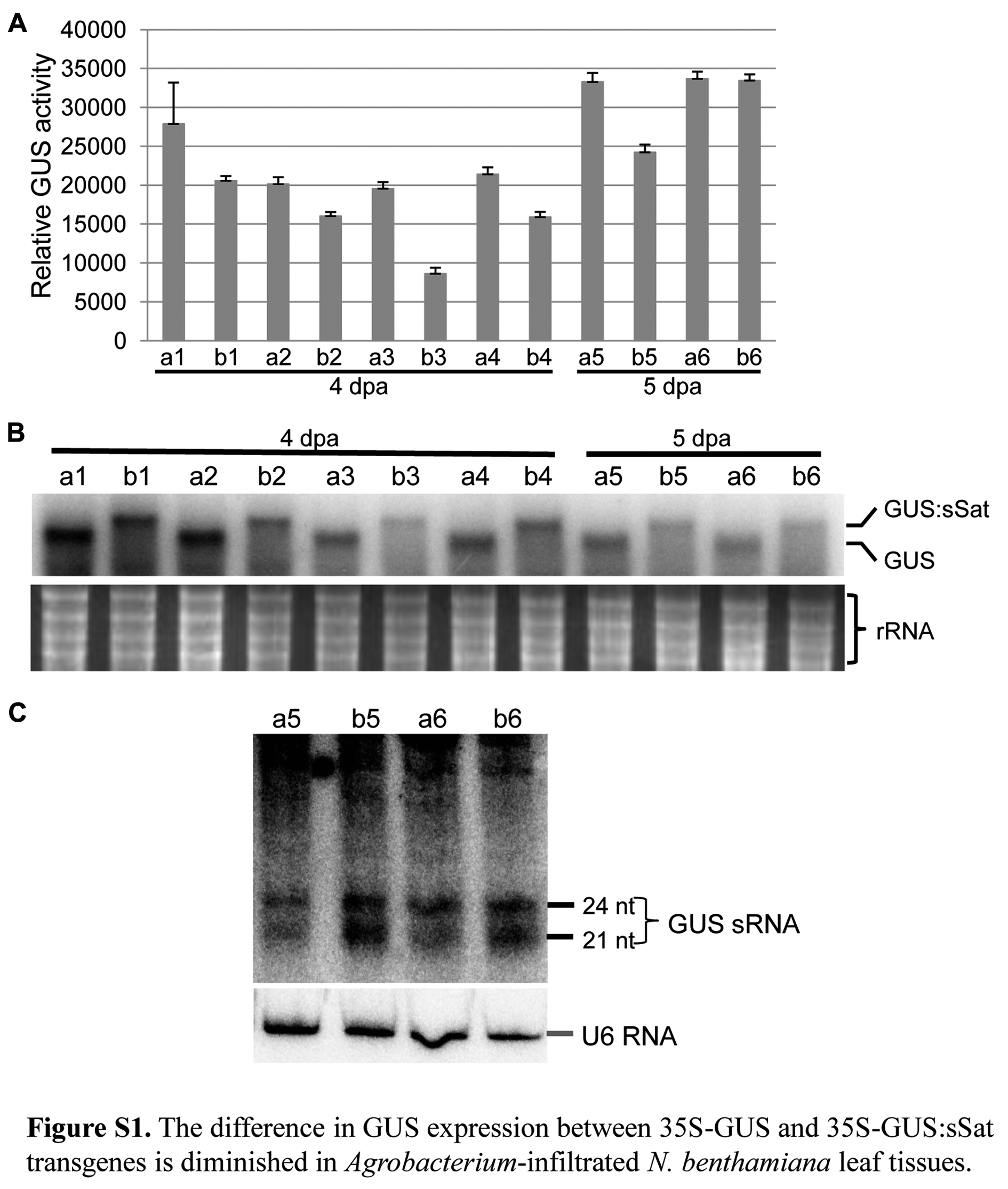

Supplement: S1 Fig — The difference in GUS expression between 35S-GUS and 35S-GUS:sSat transgenes is diminished in Agrobacterium-infiltrated N. benthamiana leaf tissues. (A) MUG assay of leaf tissues agro-infiltrated with 35S-GUS + P19 (a) or 35S-GUS:sSat + P19 (b). (B) Northern blot hybridization using antisense GUS RNA as probe. (C) Detection of small RNAs in agro-infiltrated tissues at 5 dpa (days post agro-infiltration) using northern blot hybridization. The numbers (1, 2, 3, 4, 5 and 6) denote a specific leaf of which one half was infiltrated with 35S-GUS + P19 (a) and the other half with 35S-GUS:sSat + P19 (b). Transient expression of agro-infiltrated transgenes is not expected to be affected by TGS, and PTGS (or sense cosuppression) is minimized by co-infiltrating the construct expressing P19, a strong viral suppressor of RNA silencing. Note that the difference in GUS expression between the two transgenes in this transient assay is much reduced in comparison to that in stably transformed plants (Fig. 1). The slightly lower expression level of the 35S-GUS:sSat transgene could be due to the higher level of 21-nt sRNAs shown in (C), as 21-nt sRNA-directed PTGS of the agro-infiltrated transgenes was unlikely to be completely prevented by P19. (TIF) [file pgen.1004906.s001.tif]

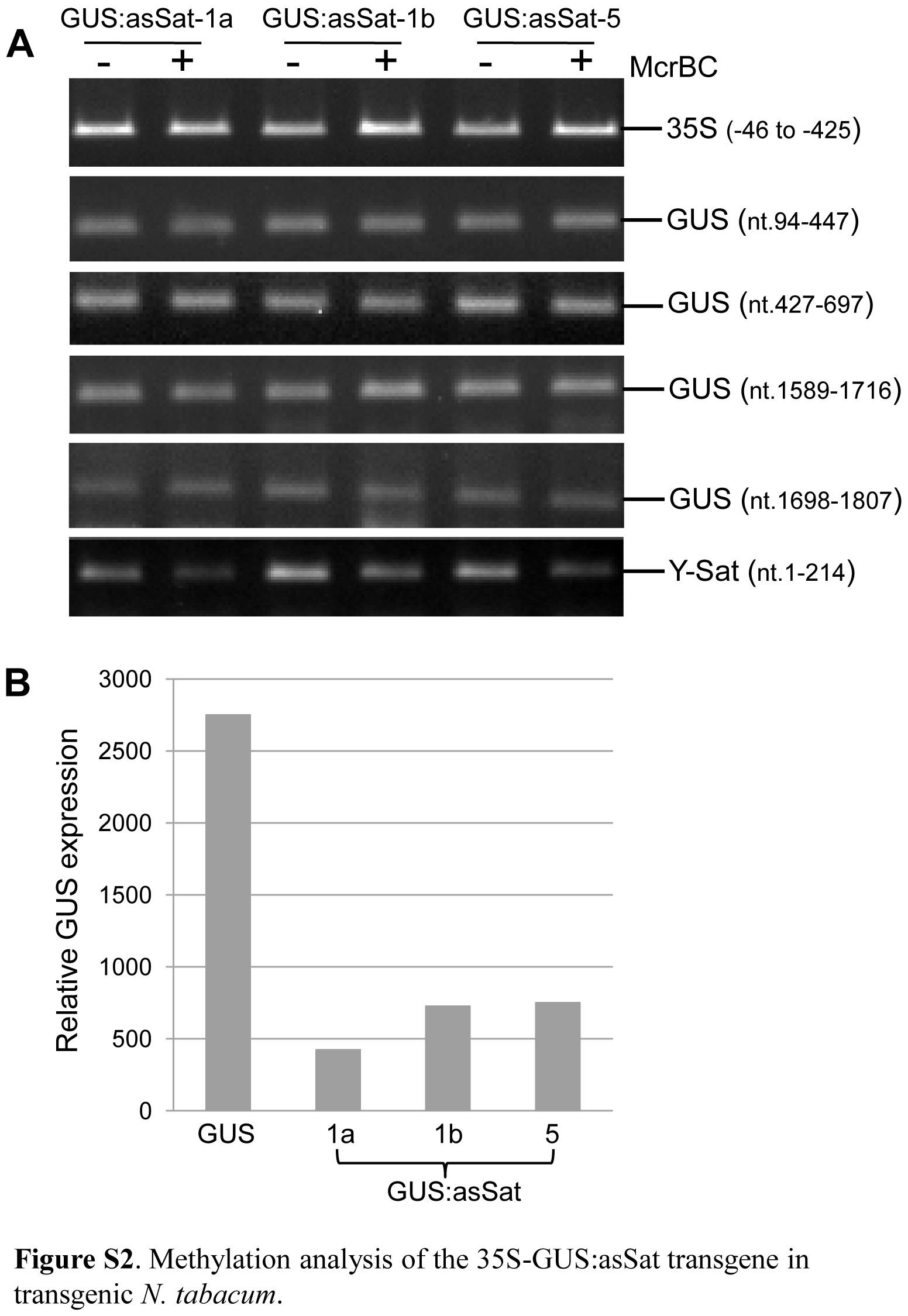

Supplement: S2 Fig — Methylation analysis of the 35S-GUS:asSat transgene in transgenic N. tabacum. (A) McrBC PCR of the 35S promoter, GUS coding and Y-Sat sequences. (B) MUG assay of GUS activity in the transgenic plants used for the McrBC PCR analysis. Note that there is no clear difference in the PCR band intensity between McrBC-digested (+) and undigested (−) samples for the 35S and GUS sequences, but there is a clear reduction in amplification for the Y-Sat sequence upon McrBC digestion. Also note that the 35S-GUS:asSat-1a plant appears to show the strongest methylation in the Y-Sat sequence, and this coincides with the lowest level of GUS expression among the three 35S-GUS:asSat plants. (TIF) [file pgen.1004906.s002.tif]

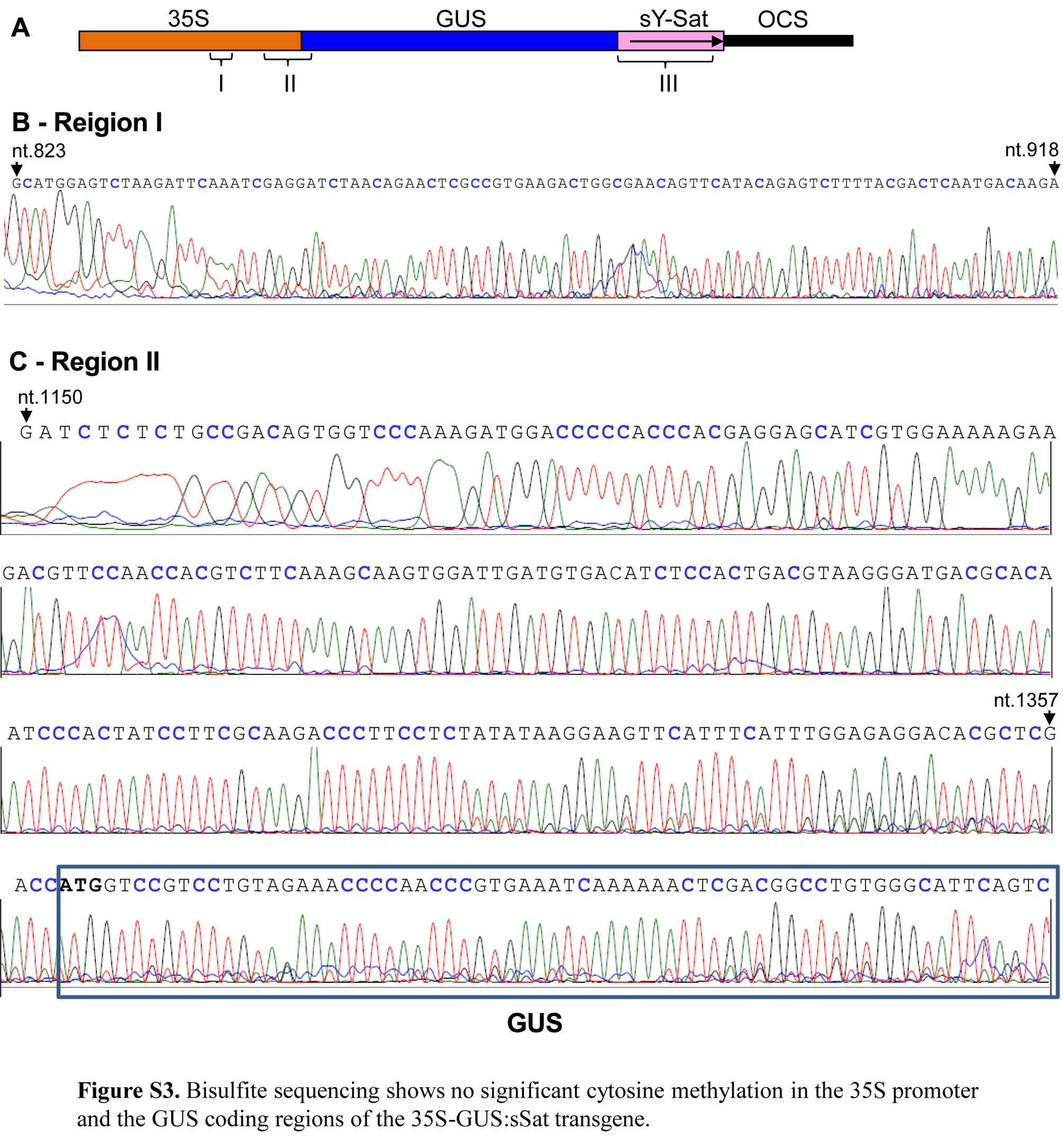

Supplement: S3 Fig — Bisulfite sequencing shows no significant cytosine methylation in the 35S promoter and the GUS coding regions of the 35S-GUS:sSat transgene. (A) The three bisulfite-sequenced regions in the 35S-GUS:sSat transgene, I, II, and III, are indicated. (B) Sequencing trace file of bisulfite PCR product from region I (35S promoter). (C) Sequencing trace file of bisulfite PCR product from region II (35S-GUS junction). The numbers in (B) and (C) indicate nucleotide positions of the bisulfite sequenced regions in the 35S promoter sequence. The GUS sequence in the 35S-GUS junction region is boxed (C). Cytosine residues of the original sequences are in bold-blue C letter. (TIF) [file pgen.1004906.s003.tif]

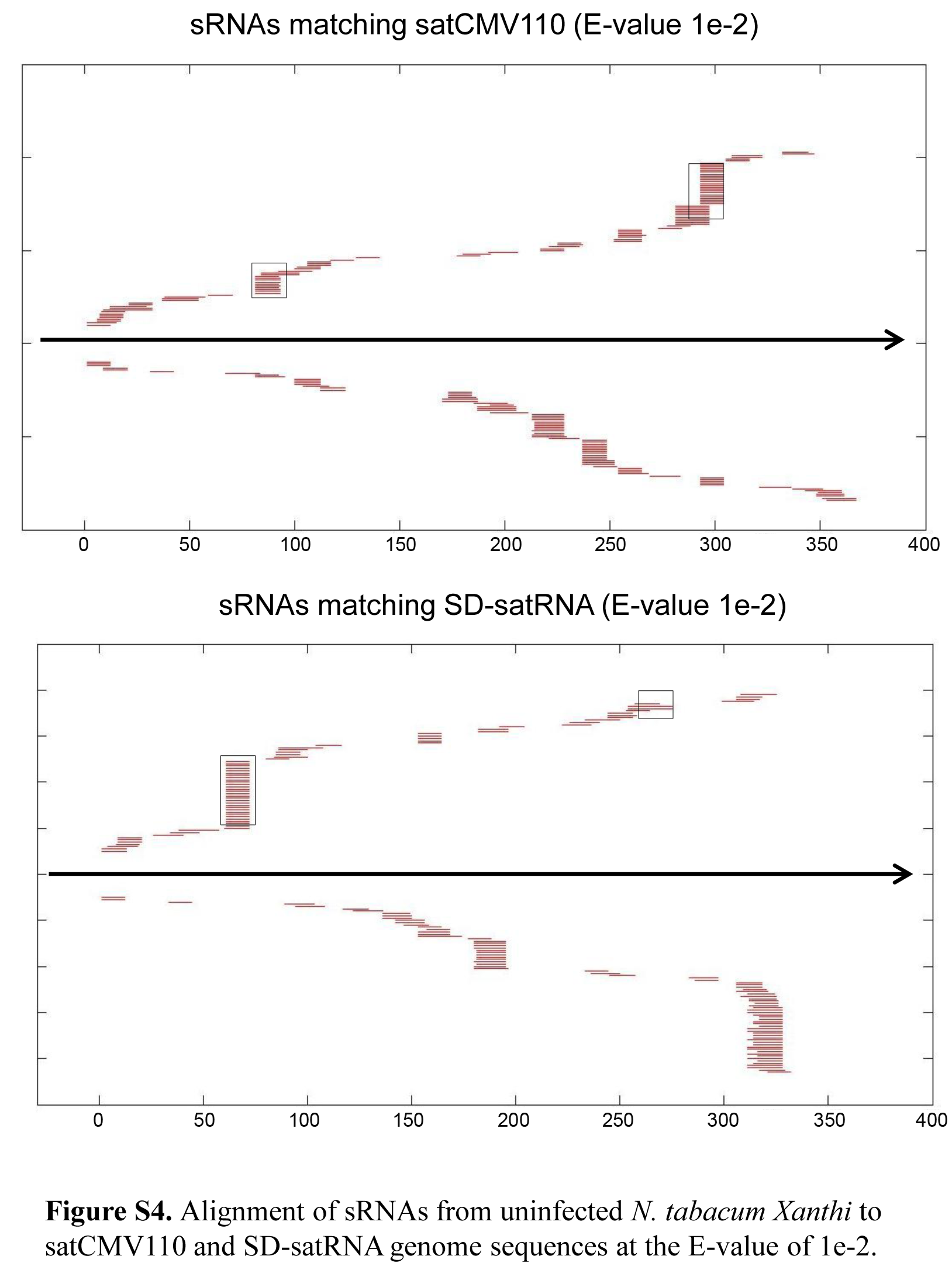

Supplement: S4 Fig — Alignment of sRNAs from uninfected N. tabacum Xanthi to satCMV110 and SD-satRNA genome sequences at the E-value of 1e−2. sRNAs matching the plus strand satRNA sequence are shown as short thin lines above the arrow-headed black lines, and those matching the minus-strand satRNA sequence shown below the arrow-headed lines. sRNAs mapped to the two most conserved regions of CMV satRNAs (positions around nt. 60–80 and nt. 280–300) are boxed. (TIF) [file pgen.1004906.s004.tif]

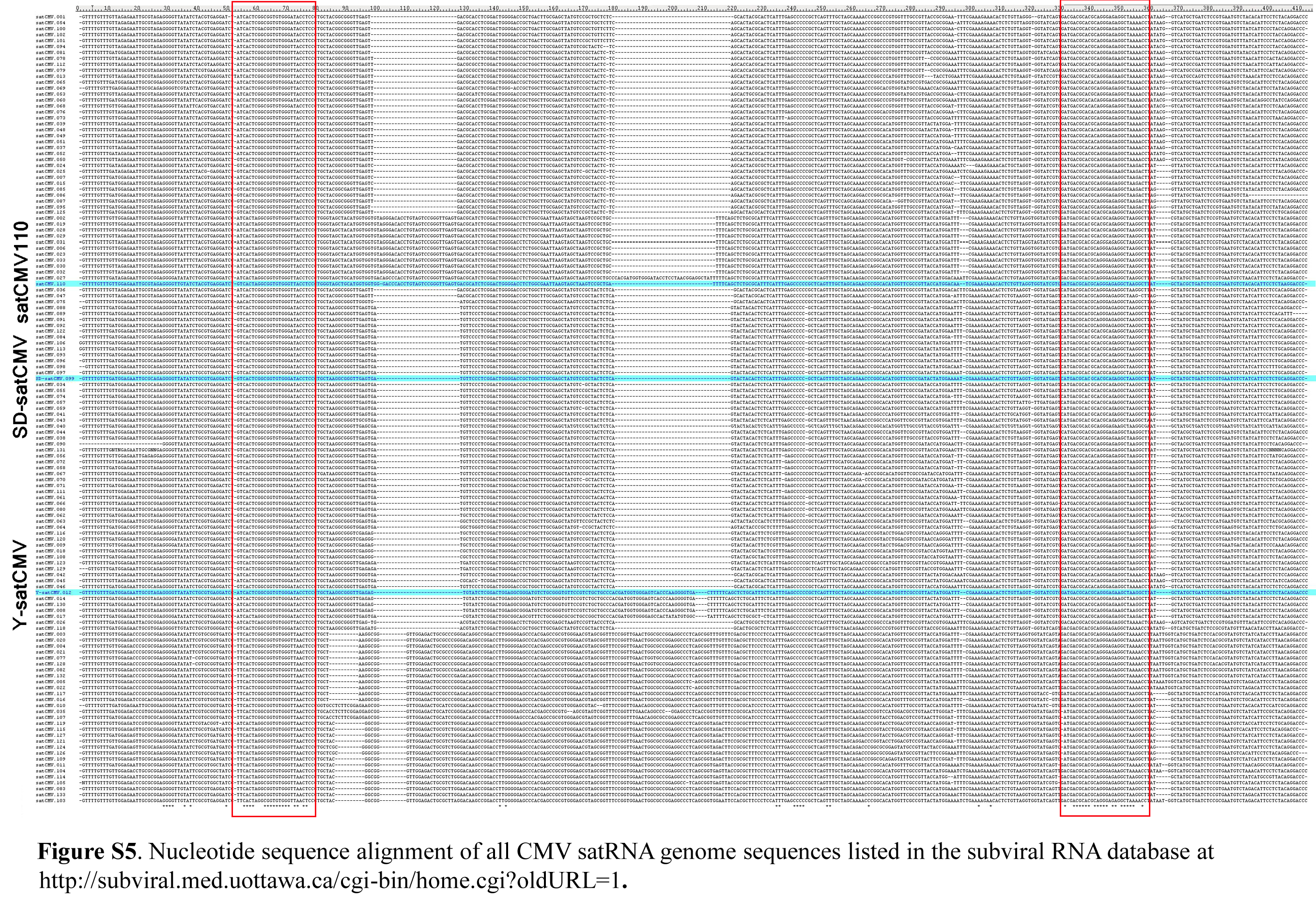

Supplement: S5 Fig — Nucleotide sequence alignment of all CMV satRNA genome sequences listed in the subviral RNA database at http://subviral.med.uottawa.ca/cgi-bin/home.cgi?oldURL=1. Sequences of SD-satRNA, Y-Sat and satCMV110 are highlighted in blue. Conserved nucleotides among all satRNAs are indicated with asterisks at the bottom. Two red boxes mark the regions corresponding to the most conserved sRNA hotspots in Fig. 6 and S4 Fig. (TIF) [file pgen.1004906.s005.tif]

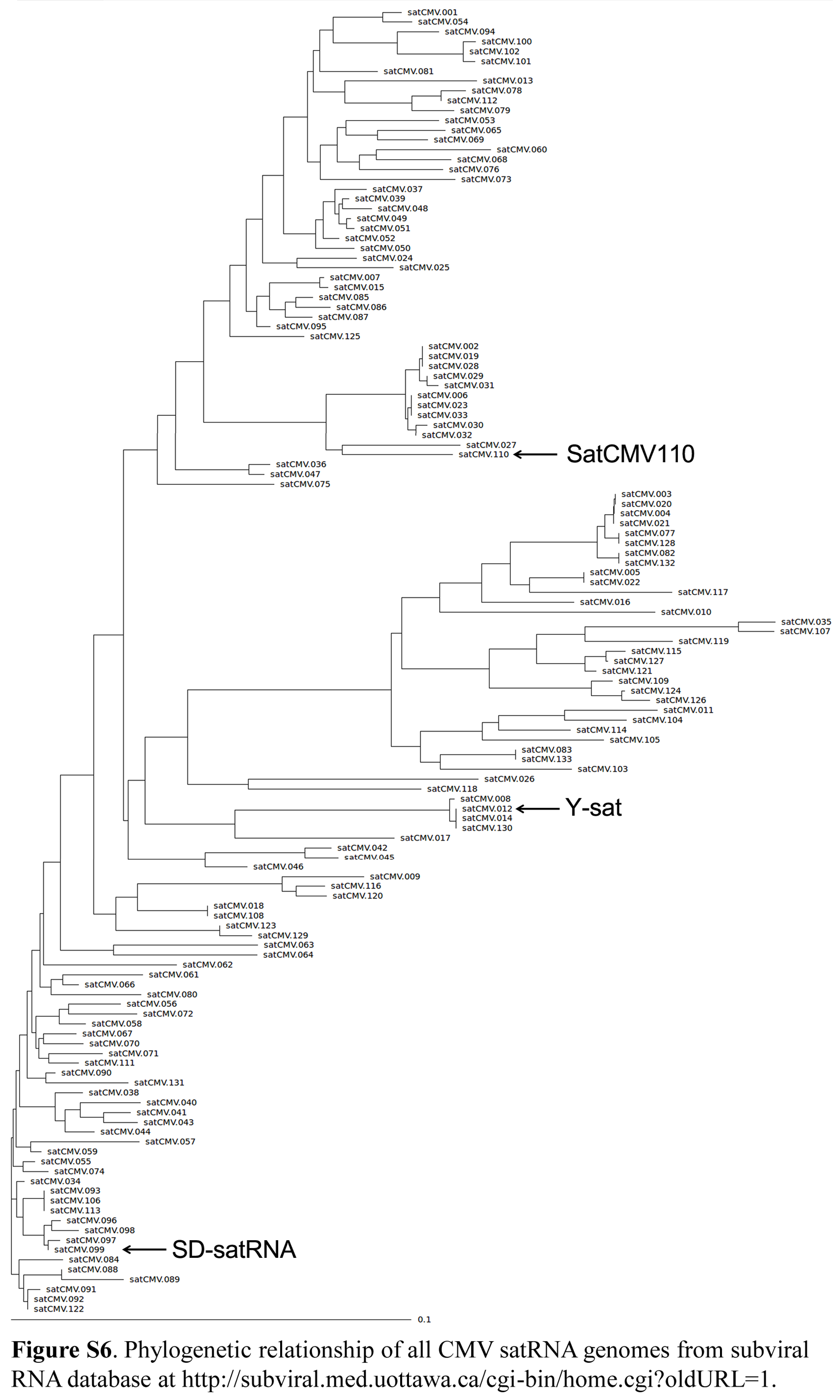

Supplement: S6 Fig — Phylogenetic relationship of all CMV satRNA genomes from subviral RNA database at http://subviral.med.uottawa.ca/cgi-bin/home.cgi?oldURL=1. Accession numbers of satRNA strains are shown. SD-satRNA, Y-Sat and satCMV110 are indicated. (TIF) [file pgen.1004906.s006.tif]
