# Supplementary material for: Nicotiana Small RNA Sequences Support a Host Genome Origin of Cucumber Mosaic Virus Satellite RNA
Source: PLoS Genet. 2015 Jan 8;11(1):e1004906. doi: 10.1371/journal.pgen.1004906 (PMC4287446; doi:10.1371/journal.pgen.1004906)
Supplement: S1 Table — A large proportion of the unique CMV satRNA-matching sRNA sequences cannot be mapped to the uncompleted N. tabacum genome. (DOCX) [file pgen.1004906.s007.docx]

**Table S1**. A large proportion of the unique CMV satRNA-matching sRNA sequences cannot be mapped to the uncompleted *N. tabacum* genome.

| Matched  sequence |  | sRNAs from uninfected *Nt-Xanthi* | | | | sRNA from SD-CMV△satRNA-infected *Nt. Xanthi* | | | | |
| --- | --- | --- | --- | --- | --- | --- | --- | --- | --- | --- |
|  | E-value | 1e-2 | 1e-3 | 1e-4 | 1e-5 | 1e-2 | 1e-3 | 1e-4 | 1e-5 |  |
| Y-Sat | Total | 170 | 148 | 40 | 0 | 1023 | 849 | 237 | 14 |  |
|  | match to *N. tabacum* genome | 75 | 64 | 4 | 0 | 331 | 275 | 44 | 2 |  |
| satCMV110 | Total | 169 | 19 | 1 | 1 | 1167 | 56 | 10 | 5 |  |
|  | match to *N. tabacum* genome | 82 | 2 | 0 | 0 | 376 | 10 | 0 | 0 |  |
| SD-satRNA | Total | 146 | 5 | 1 | 1 | 899 | 42 | 8 | 5 |  |
|  | match to *N. tabacum genome* | 37 | 0 | 0 | 0 | 203 | 5 | 1 | 0 |  |
