# Supplementary material for: Nicotiana Small RNA Sequences Support a Host Genome Origin of Cucumber Mosaic Virus Satellite RNA
Source: PLoS Genet. 2015 Jan 8;11(1):e1004906. doi: 10.1371/journal.pgen.1004906 (PMC4287446; doi:10.1371/journal.pgen.1004906)
Supplement: S2 Table — Oligonucleotide sequences. (DOCX) [file pgen.1004906.s008.docx]

**Table S2**. Oligonucleotide sequences (note: Y = C + T; R = G + A; For, forward primer; Rev, reverse primer)

| Y-satRNA genome assembly | Y-Sat1 | 5’CGCTCGAGAAGCTTGTTTTGTTTGATGGAGAATTGCGTAGAGGGGTTA |
| --- | --- | --- |
|  | Y-Sat2 | 5’AATTGCGTAGAGGGGTTATATCTGCGTGAGGATCCATCACTCGGCGGTGTGGGATACCTCCCTGCTAAGGCGGGTTGAGAGTGTATCTCGGACTGGAG |
|  | Y-Sat3 | 5’AAATGCAGAGCTGAAAAAGTCACCCCTTGGGTGACTCCCACCATCGTGGGCAGCAGACGGAACACCCGCAGACATCCCGCCTCCAGTCCGAGATA |
|  | Y-Sat4 | 5’TTCAGCTCTGCATTTCTCATTTGAGCCCCCGCTCAGTTTGCTAGCAAAACCCGGCACATGGTTCGCCGTTACTATGGATTTCGAAAGAAACACTC |
|  | Y-Sat5 | 5’ATGTATAGACATTCACGGAGATCAGCATAGCATAAGCCTTAGCTTCTCCCTGCGTGCGTCATCCACGATACCACCTAACAGAGTGTTTCTTTCGA |
|  | Y-Sat6 | 5’GGGGTACCAAGCTTGGGTCCTGTAGAGGAATGTATAGACATTCACGG |
| McrBC PCR | Y-satRNA (nt. 1-108)  Y-Sat F1  Y-Sat R1 | 5’GTTTTGTTTGATGGAGAATTG  5’TCCGAGATACACTCTCAACCC |
|  | Y-satRNA (nt.1-214; also for qPCR)  YV-F1  VAR-R1 | 5’GTTTTGTTTGTTGGAGAATTGCG  5’TGAGCGGGGGCTCAAATGAG |
|  | 35S in both Figure 3A and B  35S-F2  35S-R1 | 5’GAGGATCTAACAGAACTCGC  5’GGAACGTCTTCTTTTTCCACG |
|  | **GUS** (nt.94-445)  GUSpr8 (For)  GUSpr13 (Rev) | 5’CAGCGTTGGTGGGAAAGCGCGT  5’TCACCATTCCCGGCGGGATAG |
|  | GUS (nt.94-447)  GUSpr8 (For)  GUSpr11 (Rev) | 5’CAGCGTTGGTGGGAAAGCGCGT  5’AATCACCATTCCCGGCGGGAT |
|  | **GUS** (nt.427-697)  GUSpr10 (For)  GUSPol3-2 (Rev) | 5’ATCCCGCCGGGAATGGTGATT  5’GCGGATCCCGCTAGTGCCTTGTCC |
|  | **GUS** (nt.1589-1716)  GUSpr7 (For)  GUSpr15 (Rev) | 5’TGGATATGTATCACCGCGTC  5’AGTGAAGATCCCTTTCTTGTT |
|  | **GUS** (nt.1698-1787)  GUSpr14 (For)  GUSpr17 (Rev) | 5’CAAGAAAGGGATCTTCACTCG  5’GTTTGCCTCCCTGCTGCGGTT |
| Bisulfite Region I | 35S-BisFa  35S-BisFb (nested primer)  35S-BisRc  35S-BisRd (nested primer) | 5’AGGYAAGTAATAGAGATTGGAG  5’GATTGGAGTYTYTAAAAAGGTAG  5’TTCTTTTTCCACRATRCTCCTC  5’CTTRAATRATARCCTTTCCTTTATC |
| Bisulfite Region II | Bis35SF1  Bis35SF2 (nested primer)  BisGUS-FLR1  BisGUS-FLR2 (nested primer) | 5’GAYAGTAGAAAAGGAAGGTGG  5’ATYATTGYGATAAAGGAAAGG  5’CTTTCTTRTAACRCRCTTTCC  5’TAACRCRCTTTCCCACCAAC |
| Bisulfite Region III | Bis-GUS-F4  Bis-GUS-F2 (nested primer)  Bis-Sat-NR1  Bis-Sat-NR2 (nested primer) | 5’GYAAAYYGAAGTYTTGYGG  5’TGTAGTAGGGAGGTAAATAATGAAT  5’CATAARCCTTARCTTCCCCC  5’CRTCATCCACRATACCACC |
| Nuclear run-on probes | **For amplifying GUS**:  M13-F  M13-R  **For amplifying EF1α**:  EF1α-F  EF1α-R  **For amplifying FKS1**:  FKS1-F  FKS1-R | 5’GTAAAACGACGGCCAGT  5’AACAGCTATGACCATG  5’GGTGACAACATGCTCGAAAG  5’GGATCCTTGCAACAACCATG  5`TTTGATCTATTTCTGGATC  5`GTTGGCAGTGTCGCCCTTG |
