# Supplementary material for: Correction: Nicotiana Small RNA Sequences Support a Host Genome Origin of Cucumber Mosaic Virus Satellite RNA
Source: PLoS Genet. 2015 Apr 27;11(4):e1005196. doi: 10.1371/journal.pgen.1005196 (PMC4411101; doi:10.1371/journal.pgen.1005196)
Supplement: S1 File — (PDF) [file pgen.1005196.s001.pdf]

PLOS | GENETICS

Nicotiana Small RNA Sequences Support a Host Genome Origin of *Cucumber Mosaic Virus* Satellite RNA

Kiran Zahid[1,2,¤], Jian-Hua Zhao[3], Neil A. Smith[1], Ulrike Schumann[1], Yuan-Yuan Fang[3], Elizabeth S. Dennis[1], Ren Zhang[2], Hui-Shan Guo[3]*, Ming-Bo Wang[1]*

1 CSIRO Plant Industry, Canberra, Australian Capital Territory, Australia, **2** School of Biological Sciences, University of Wollongong, Wollongong, New South Wales, Australia, **3** State Key Laboratory of Plant Genomics and National Center for Plant Gene Research, Institute of Microbiology, Chinese Academy of Sciences, Beijing, China

Abstract

Satellite RNAs (satRNAs) are small noncoding subviral RNA pathogens in plants that depend on helper viruses for replication and spread. Despite many decades of research, the origin of satRNAs remains unknown. In this study we show that a β-glucuronidase (GUS) transgene fused with a *Cucumber mosaic virus* (CMV) Y satellite RNA (Y-Sat) sequence (35S-GUS:Sat) was transcriptionally repressed in *N. tabacum* in comparison to a 35S-GUS transgene that did not contain the Y-Sat sequence. This repression was not due to DNA methylation at the 35S promoter, but was associated with specific DNA methylation at the Y-Sat sequence. Both northern blot hybridization and small RNA deep sequencing detected 24-nt siRNAs in wild-type *Nicotiana* plants with sequence homology to Y-Sat, suggesting that the *N. tabacum* genome contains Y-Sat-like sequences that give rise to 24-nt sRNAs capable of guiding RNA-directed DNA methylation (RdDM) to the Y-Sat sequence in the 35S-GUS:Sat transgene. Consistent with this, Southern blot hybridization detected multiple DNA bands in *Nicotiana* plants that had sequence homology to Y-Sat, suggesting that Y-Sat-like sequences exist in the *Nicotiana* genome as repetitive DNA, a DNA feature associated with 24-nt sRNAs. Our results point to a host genome origin for CMV satRNAs, and suggest novel approach of using small RNA sequences for finding the origin of other satRNAs.

Citation: Zahid K, Zhao JH, Smith NA, Schumann U, Fang YY, et al. (2014) *Nicotiana* Small RNA Sequences Support a Host Genome Origin of *Cucumber Mosaic Virus* Satellite RNA. PLoS Genet 11(1): e1004906. doi:10.1371/journal.pgen.1004906

Editor: Xuemei Chen, University of California Riverside, United States of America

Received June 20, 2014; **Accepted** November 20, 2014; **Published** January 8, 2015

Copyright: © 2014 Zahid et al. This is an open-access article distributed under the terms of the Creative Commons Attribution License, which permits unrestricted use, distribution, and reproduction in any medium, provided the original author and source are credited.

Data Availability: The authors confirm that all data underlying the findings are fully available without restriction. All data are within the paper except the raw small RNA sequencing data which are available from NCBI under accession number GSE63586.

Funding: This work was supported by an Australian Research Council Future Fellowship (FT0991956; MBW) and grants from the Ministry of Science and Technology (2014CB138402) and the Natural Science Foundation of China (No. 91219301; HSG). The funders had no role in study design, data collection and analysis, decision to publish, or preparation of the manuscript.

Competing Interests: The authors have declared that no competing interests exist.

* Email: ming-bo.wang@csiro.au (MBW); guohs@im.ac.cn (HSG)

These authors contributed equally to this work.

¤ Current address: Atta-ur-Rahman School of Applied Biosciences, National University of Sciences and Technology, Islamabad, Pakistan

Introduction

Satellite RNAs (satRNAs) are among the smallest RNA pathogens in plants and depend on associated viruses (helper viruses) for replication, encapsidation and movement inside the host plant [1,2]. Their RNA genomes range from 220 to 1500 nucleotides (nt) in size and can form compact secondary structures by intra-molecular base-pairing that can be resistant to degradation by ribonucleases. SatRNAs are classified into three classes [3]. Class 1 satRNAs include large mRNA satellites that are 800 to 1500 nt in length and contain a single open reading frame that encodes at least one non-structural protein. SatRNAs belonging to class 2 are linear, less than 700 nt in size and possess no mRNA activity so do not encode any protein. SatRNAs of this class, including the *Cucumber mosaic virus* (CMV) satRNAs [4], occur most frequently. SatRNAs of class 3 are circular, around 350 to 400 nt in length and also do not exhibit mRNA activity. SatRNAs normally accumulate at high levels in infected host plants relative to their helper viruses, presumably because of the small size and ribonuclease-resistant structure of their RNA genome. A previous study shows that a CMV satRNA, unlike the CMV helper virus, is resistant to host RNA-dependent RNA polymerase-mediated antiviral silencing in Arabidopsis [5], which may also contribute to the high level accumulation of satRNAs. Whereas high-level replication and systemic infection of satRNAs depend on helper virus-encoded proteins, recent studies on CMV satRNAs indicate that satRNAs can be imported into the nucleus and transcribed there by host plant proteins independently of helper viruses [6,7]. satRNAs are not required for the life cycle of their helper viruses, but participate in helper virus-host interactions by modulating the level of helper virus accumulation and the severity of helper virus-induced symptoms [8]. In addition, satRNAs can induce disease symptoms in the host plants that are distinct from helper virus-caused symptoms [4]. Recent studies indicate that such satRNA-induced symptoms are due to silencing of host genes directed by satRNA-derived small interfering RNAs (siRNA) [9,10].

Like all plant viruses and subviral agents, the origin of satRNAs remains unclear. Two main origins of satRNA have been suggested: the genome of the helper virus or that of the host plant. However, unlike defective interfering RNAs, a

Author Summary

Satellite RNAs (satRNAs) are small RNA pathogens in plants that depend on associated viruses for replication and spread. While much is known about the replication and pathogenicity of satRNAs, their origin remains a mystery. We report evidence for a host genome origin of the *Cucumber mosaic virus* (CMV) satRNA. We show that only the CMV Y-satRNA (Y-Sat) sequence region of a fusion transgene was methylated in *Nicotiana tabacum*, indicating that the Y-Sat sequence is subject to 24-nt small RNA (sRNA)-directed DNA methylation. 24-nt sRNAs as well as multiple genomic DNA fragments, with sequence homology to Y-Sat, were detected in *Nicotiana* plants, suggesting that the *Nicotiana* genome contains Y-Sat-like repetitive DNA sequences, a genomic feature associated with 24-nt sRNAs. Our results suggest that CMV satRNAs have originated from repetitive DNA in the *Nicotiana* plant genome, and highlight the possibility that small RNA sequences can be used to identify the origin of other satRNAs.

group of subviral RNAs derived from truncated forms of the helper virus genome, satRNAs usually possess little or no sequence homology with their helper viruses [1], which argues against the helper virus genome as their origin. One exception is the virulent satRNA strain of *Turnip crinkle virus*, which contains a long (166-nt) segment that is homologous to the 3' end of the helper virus genome [11]. A number of studies have suggested satRNA emergence from the host genome. Sequence similarity has been observed between nucleotide stretches of the *Arabidopsis* genome and CMV satRNAs [1]. SatRNAs, such as CMV satRNAs that occur widely in *Nicotiana* species and some other *Solanaceae* species, are more commonly detected in experimental systems than in the wild or nature [1]. A number of studies have reported *de novo* emergence of satRNAs on serial passaging plants with the helper virus under controlled environmental conditions [12], implicating the host genome as the origin of satRNAs. Another report showed structural similarities of *Peanut stunt virus* (PSV) satRNAs with cellular introns of nucleus, mitochondria and plant viroids [13]. However, in spite of these suggestions, no intact plant genome sequence with similarities to satRNAs has been reported.

RNA silencing is an evolutionarily conserved gene regulation mechanism in eukaryotes mediated by 20-25-nt small RNAs (sRNAs) [14,15]. These sRNA are processed from double-stranded (ds) or hairpin (hp) RNA by Dicer or Dicer-like (DCL) protein. To induce silencing, one strand of a sRNA is loaded into an Argonaute (AGO) protein to form the RNA-induced silencing complex (RISC), and guides the RISC to bind to complementary single-stranded RNA and cleave the RNA. Plants have three basic types of sRNA, 20-24-nt microRNA (miRNA), 21-22-nt siRNA, and 24-nt repeat-associated siRNA (rasiRNA) [16,17]. MiRNAs induce posttranscriptional degradation or translational repression of mRNAs that encode regulatory proteins, such as transcription factors, and play a key role in plant development [17,18]. The 21-22-nt siRNAs direct degradation of viral RNA and some endogenous mRNA, and are important in plant defence against viruses and in the control of some endogenous genes [17,19]. The 24-nt rasiRNAs are unique to plants, and are involved in RNA-directed DNA methylation (RdDM) which is important for maintaining genome stability by silencing transposons and repetitive DNA sequences [17,20,21]. RdDM is highly sequence specific, and can be induced by both endogenous siRNAs and siRNAs derived from infecting viral agents including satRNAs [20,22].

During the analysis of a transgene (35S-GUS:Sat) containing a β-glucuronidase (GUS) sequence transcriptionally fused at the 3′ end with the CMV Y-satellite RNA (Y-Sat) sequence in *Nicotiana tabacum*, we observed that the Y-Sat sequence was specifically methylated. This led us to hypothesize that 24-nt siRNAs homologous to Y-Sat may exist in *N. tabacum* inducing RdDM at the Y-Sat sequence of the transgene. Subsequent analyses revealed the existence of both 24-nt sRNAs and multiple DNA fragments in *Nicotiana* plants that showed sequence homology to Y-Sat, suggesting that CMV satRNAs originate from repetitive regions in the *Nicotiana* genome.

Results

35S-GUS:Sat transgenes are repressed in transgenic tobacco

Three 35S promoter-driven GUS constructs were created, two of which had a 3′ fusion of a full-length 369-nt Y-Sat sequence [23] in either the sense (sSat) or antisense (asSat) orientation (Fig. 1A). These were transformed into tobacco and multiple independent transgenic lines obtained for each.

Plants transformed with the 35S-GUS:Sat fusion constructs showed reduced levels of GUS protein in comparison to those transformed with just the 35S-GUS construct lacking the Y-Sat sequence (Fig. 1B, 1C). This reduction in GUS activity occurred for both the sense and antisense orientations of the Y-Sat sequence (Fig. 1B). The low level of GUS activity was relatively uniform across the independent primary (T0) transformants (Fig. 1B) and persisted in the subsequent (T1 and T2) generations (Fig. 1C). The MUG assay results were confirmed by northern blot hybridization, which showed that the GUS:sSat and GUS:asSat transcripts accumulated at a much lower level than the GUS transcript in the respective transgenic plants (Fig. 1D).

The reduced expression of the 35S-GUS:sSat transgene is due to transcriptional repression

The repressed expression of the 35S-GUS:Sat transgenes could either be due to transcriptional (TGS) or posttranscriptional (PTGS) gene silencing or to transcript instability caused by the fused Y-Sat sequence. Since both the 35S-GUS:sSat and 35S-GUS:asSat transgenes showed similar reduction in expression, the secondary structures of the fusion sequence were not likely to be responsible for the repression, as sense and antisense Y-Sat sequences are predicted to form different secondary structures. It also implied that RNA instability was not the main cause for the repressed GUS:Sat trangene expression. This was supported by results from an *Agrobacterium* infiltration (agro-infiltration) assay where both TGS and PTGS were negated, which showed that the large difference in expression levels between the 35S-GUS and 35S-GUS:sSat transgenes observed in stably transformed plants (~6 fold; Fig. 1) was dramatically reduced in the agro-infiltrated tissues (~1.0–2.4 fold; S1 Fig.).

We next investigated if PTGS or TGS was responsible for the repressed expression of the 35S-GUS:Sat transgenes. PTGS of a transgene is associated with 21-nt siRNAs corresponding to the transcribed region [17]. Northern blot hybridization failed to detect GUS- or Y-Sat-specific 21-nt siRNAs in three of the four 35S-GUS:Sat transgenic lines analysed (Fig. 2A), suggesting that TGS, but not PTGS, was

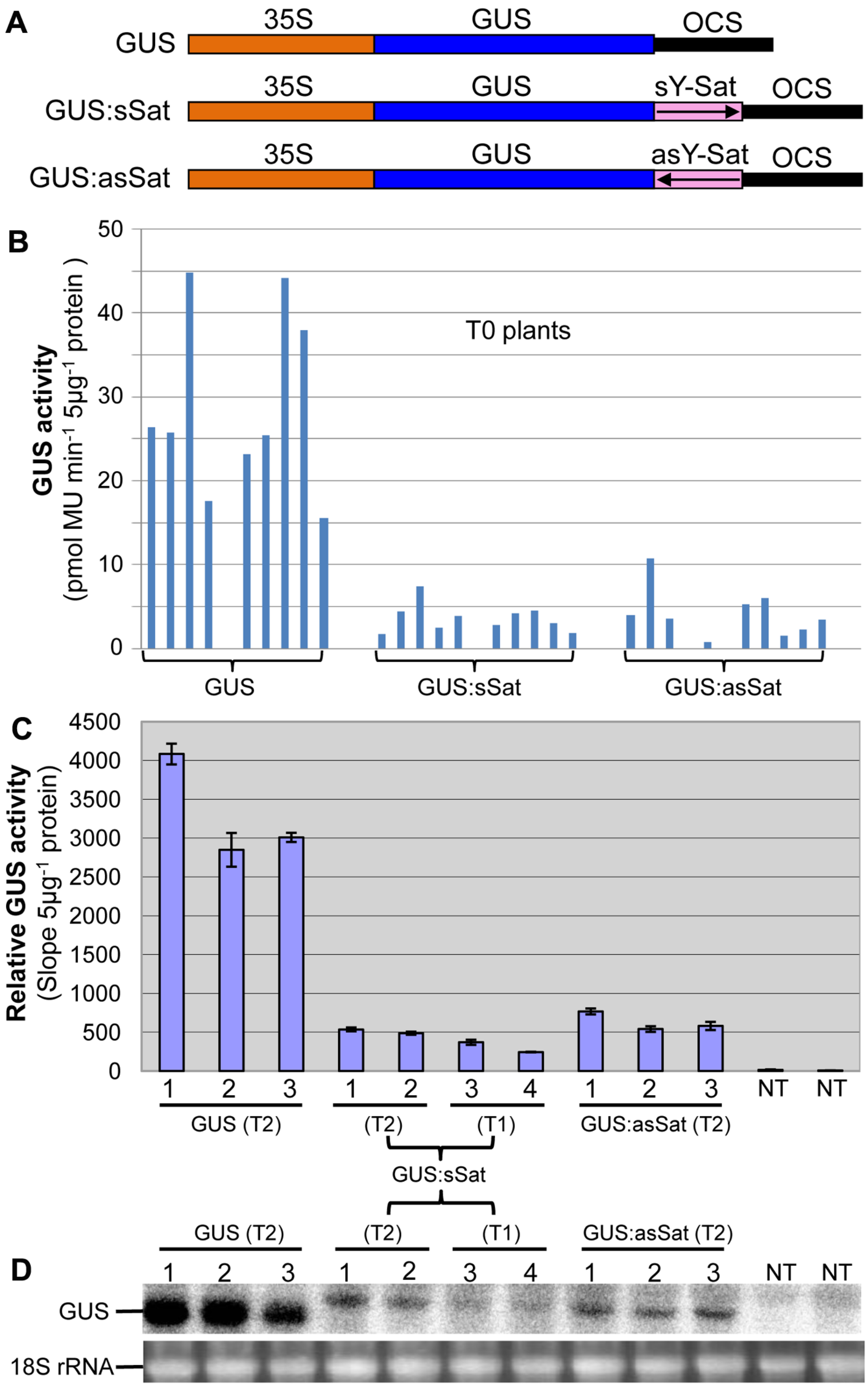

Fig. 1. The 35S-GUS:Sat fusion transgenes show repressed expression in *N. tabacum* in comparison to the 35S-GUS transgene. (A) Schematic diagrams of the transgene constructs. (B) MUG assay of independent primary (T0) transformants. (C) MUG assay of second (T1) and third-generation (T2) transformants, plus non-transgenic (NT) control plants. (D) mRNA northern blot analysis of the plants shown in (C). Note: the different units in the Y-axis of the two MUG assay histograms are due to the use of different fluoroscan machines and different ways of calculation.
doi:10.1371/journal.pgen.1004906.g001

the main cause of transgene repression. Consistent with this, nuclear run-on assay showed that the repressed 35S-GUS:sSat and 35S-GUS:asSat transgenes generated much reduced RNA signals in comparison to the highly expressed 35S-GUS transgene (Fig. 2B, C), indicating that they are transcriptionally repressed.

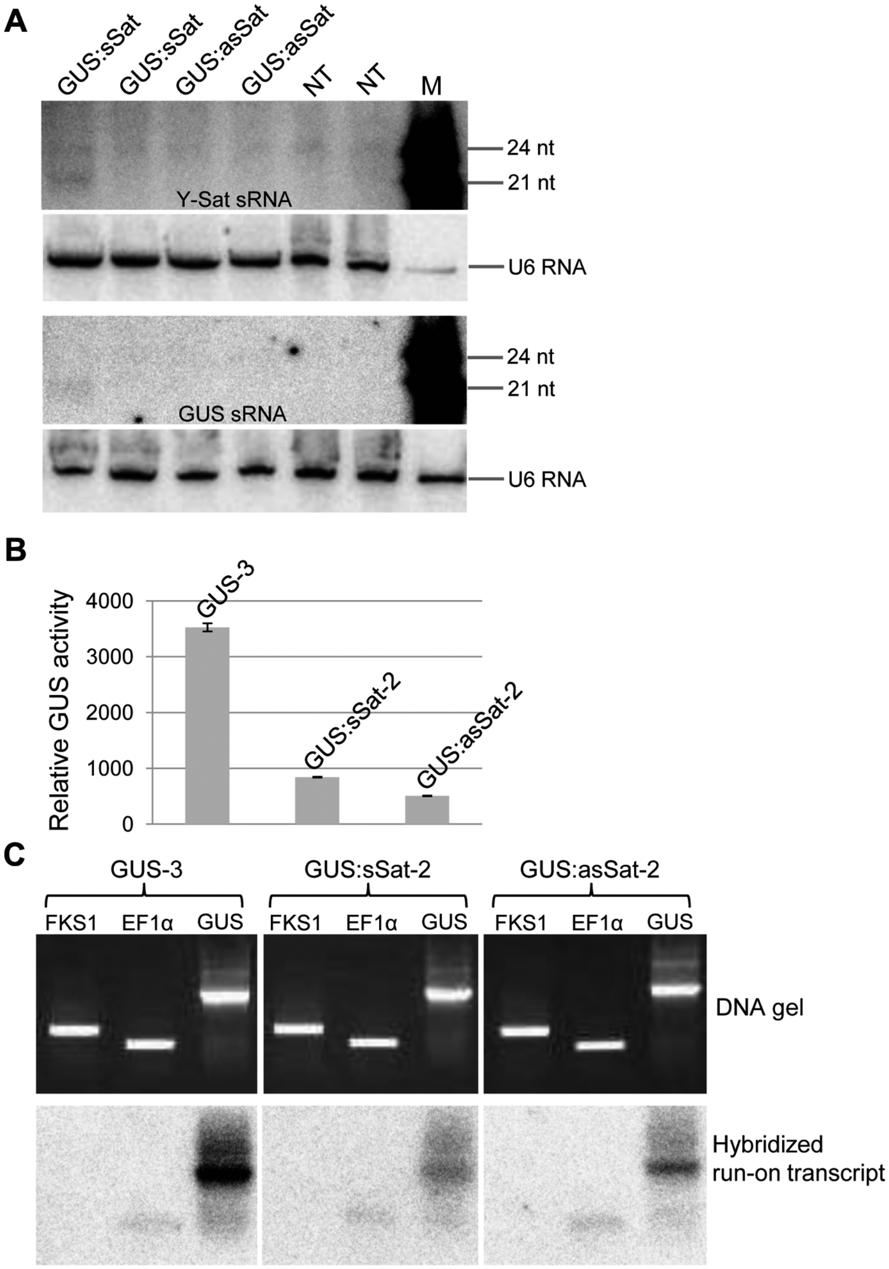

Fig. 2. The 35S-GUS:Sat transgene is transcriptionally repressed. (**A**) Three of the four 35S-GUS:Sat transgenic lines analysed show no detectable accumulation of 21-nt PTGS-associated GUS or Y-Sat-specific siRNAs, indicating that PTGS is not the main cause of 35S-GUS:Sat transgene repression. M, 21 and 24-nt small RNA size markers. (**B**) and (**C**) The highly expressed 35S-GUS line (GUS-3) (B) generates much stronger nuclear run-on transcript signals than the two repressed 35S-GUS:Sat lines (C), indicating that the reduced expression of the 35S-GUS:Sat transgenes results from transcriptional repression. FKS1 and EF1α are negative control and internal reference gene sequences, respectively.
doi:10.1371/journal.pgen.1004906.g002

The Y-Sat DNA sequence in the 35S-GUS:Sat fusion transgenes is specifically methylated in transgenic *N. tabacum*

As DNA methylation at promoter sequences can cause TGS [24], we investigated if DNA methylation occurred in the 35S promoter of the 35S-GUS:sSat transgene using McrBC digestion-PCR. McrBC is a methylation-dependent restriction enzyme that recognizes DNA containing two or more methylated cytosine residues, separated by 30–2000 base pairs, and cleaves the DNA at multiple sites close to one of the methylated cytosines. Differences in PCR-amplified McrBC-digested and undigested DNA can provide a measurement of DNA methylation levels. McrBC digestion did not result in clear reduction in the amplification of a 380-bp amplicon, derived from the the 35S promoter near the transcription start site, which contains a total of 175 cytosines from both strands (Fig. 3). This indicated that the 35S promoter in the 35S-GUS:sSat transgene was not methylated, and that the reduced transgene expression was not due to promoter methylation. We extended the DNA methylation analysis to the GUS and Y-Sat sequence of the transgene. Similar to the 35S promoter sequence, four different regions of the GUS coding sequence

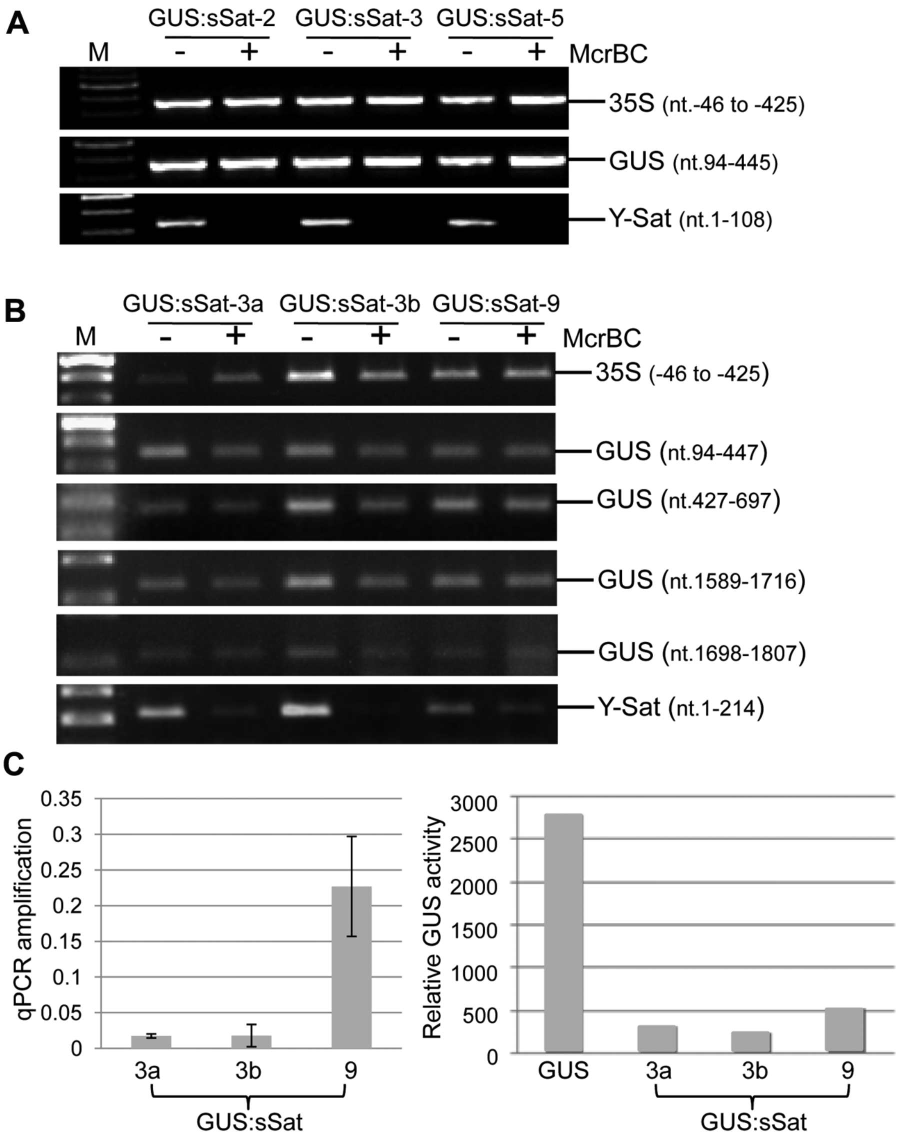

Fig. 3. Methylation analysis of the 35S-GUS:sSat transgene in transgenic *N. tabacum* using McrBC PCR. (A) and (B) McrBC PCR of 35S promoter, different regions of GUS coding sequence, and Y-Sat. (C) qPCR quantification of McrBC digestion of the Y-Sat sequence shown in (B) (nt. 1-214) (left) and MUG assay of GUS activity in the transgenic plants used for the McrBC PCR analysis (right). M, DNA size marker.
doi:10.1371/journal.pgen.1004906.g003

showed no clear methylation, as indicated by strong amplification of McrBC-digested DNA (Fig. 3).

In contrast to the 35S and GUS sequences, McrBC digestion strongly reduced PCR amplification of the Y-Sat sequence in the 35S-GUS:sSat transgene (Fig. 3), indicating that it was highly methylated. Furthermore, the degree of this methylation, as judged by the extent of reduction in PCR amplification upon McrBC digestion (Fig. 3B and C, left), appeared to be inversely correlated with the level of GUS activity in the 35S-GUS:sSat plants (Fig. 3C, right). McrBC PCR also indicated Y-Sat-specific DNA methylation in the 35S-GUS:asSat transgene that contains an antisense Y-Sat sequence (S2 Fig.). Both the 35S and GUS sequences showed no difference in PCR amplification between McrBC-digested and undigested DNA, whereas the Y-Sat sequence showed a clear reduction in amplification upon McrBC digestion. Furthermore, the expression level of the 35S-GUS:asSat transgene also appeared to be inversely correlated with the extent of Y-Sat sequence methylation (S2 Fig.).

The McrBC PCR results were validated using bisulfite sequencing, which determines DNA methylation at a single cytosine nucleotide level due to the ability of bisulfite to convert unmethylated, but not methylated, cytosines to uracils [25]. Three regions of the 35S-GUS:sSat transgene were amplified from bisulfite-converted DNA, including the 35S promoter, the 35S-GUS junction, and the Y-Sat sequence (S3A Fig.). We sequenced the bisulfite PCR product as a mixed DNA population, and determined the DNA methylation level based on the ratio between the peak heights of cytosine (C) and thymine (T) residues in the sequencing trace files, which has proven to be an effective way for measuring overall DNA methylation levels in a specific plant sample [26]. Consistent with the McrBC PCR result, the 35S and GUS sequences showed no significant methylation of cytosine residues as indicated by the lack of cytosine (blue) peaks at the cytosine positions in the trace files, which were instead replaced by thymine (red) peaks (S3B-C Fig.). In contrast, the Y-Sat sequence showed strong methylation in all four DNA samples analyzed,

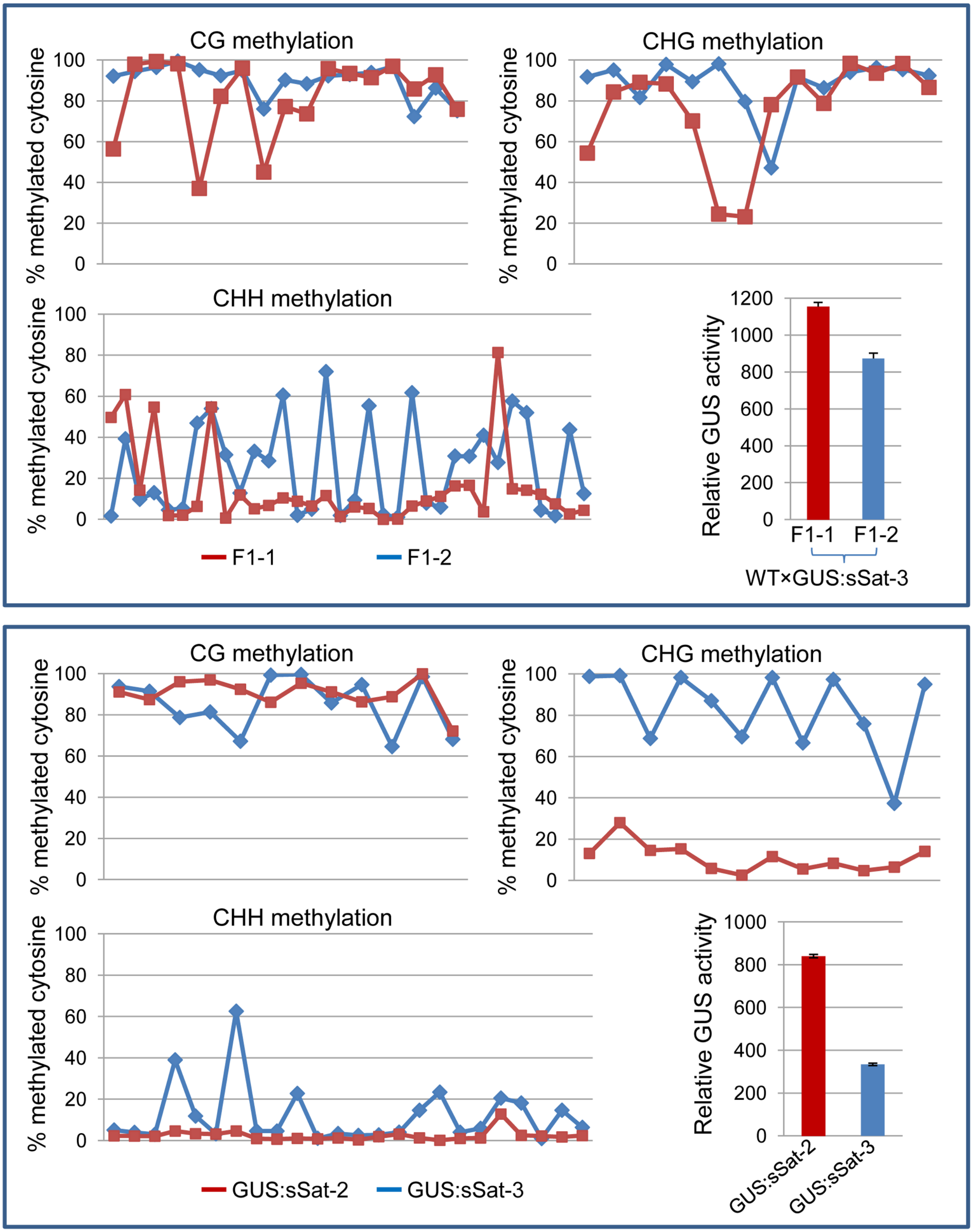

Fig. 4. Bisulfite sequencing shows strong DNA methylation in the Y-Sat sequence of the 35S-GUS:sSat transgene in all cytosine contexts, particularly at the CG and CHG sites. The methylation status of cytosines in CG, CHG and CHH contexts is presented separately in three line graphs. Each point on the line represents a cytosine in a sequential 5′ to 3′ order along the bisulfite-sequenced Y-Sat sequence, with the Y-axis showing the percentage of methylated cytosines (based on the ratio of cytosine peak height in the trace file) at each of these positions. The top panel shows the methylation pattern of two F1 sibling plants derived from a cross between WT *N. tabacum* (as the maternal parent) and a 35S-GUS:sSat transgenic line. The bottom panel shows the methylation pattern of two independent 35S-GUS:sSat transgenic lines.

Note that in both cases the plant showing a higher level of GUS expression (F1-1 or GUS:sSat-2, shown in red) displays a lower degree of CHG and CHH methylation in the Y-Sat sequence.
doi:10.1371/journal.pgen.1004906.g004

especially at the CG and CHG sites (H = A, C or T nucleotides) (Fig. 4). Two pairs of 35S-GUS:sSat transgenic lines were analyzed by bisulfite sequencing, and in each pair the plants that showed lower GUS expression level displayed a higher degree of cytosine methylation, particularly at the CHG and CHH sites (Fig. 4). Taken together, the DNA methylation analyses indicated that the Y-Sat sequence of the 35S-GUS:Sat transgenes was specifically targeted for methylation in transgenic *N. tabacum* plants, and that this methylation appeared to correlate with the repression of the transgenes.

Y-Sat-like 24-nt sRNAs are detected in *Nicotiana* plants using northern blot hybridization

The sequence-specific DNA methylation detected in the Y-Sat sequence of the 35S-GUS:Sat transgenes raised the possibility that the Y-Sat sequence might be subject to RNA-directed DNA methylation (RdDM). RdDM can occur at all cytosine contexts (CG, CHG and CHH), and is directed by the 24-nt size class of siRNAs [17,20,21]. sRNAs of 24 nt, but not of 21–22 nt, were readily detectable in both transgenic and wild-type *Nicotiana* plants by northern blot hybridization using the Y-Sat sequence as a probe, especially in the flowers (Fig. 5A), a tissue known to contain relatively high abundance of 24-nt siRNAs [27,28]. Importantly, the 24-nt sRNA signals were not affected by the presence of the 35S-GUS:Sat fusion transgenes (Fig. 5A and 2A), indicating that they are generated by the host plant genome and not by the transgene. Northern blot hybridization also showed that these 24-nt Y-Sat-like sRNAs are present in all three *Nicotiana* species analysed (Fig. 5B). These results indicated that *Nicotiana* species generate 24-nt sRNAs with sequence homology to the Y-Sat, and suggested that the DNA methylation of the Y-Sat sequence in the fusion transgenes was likely induced by these 24-nt sRNAs.

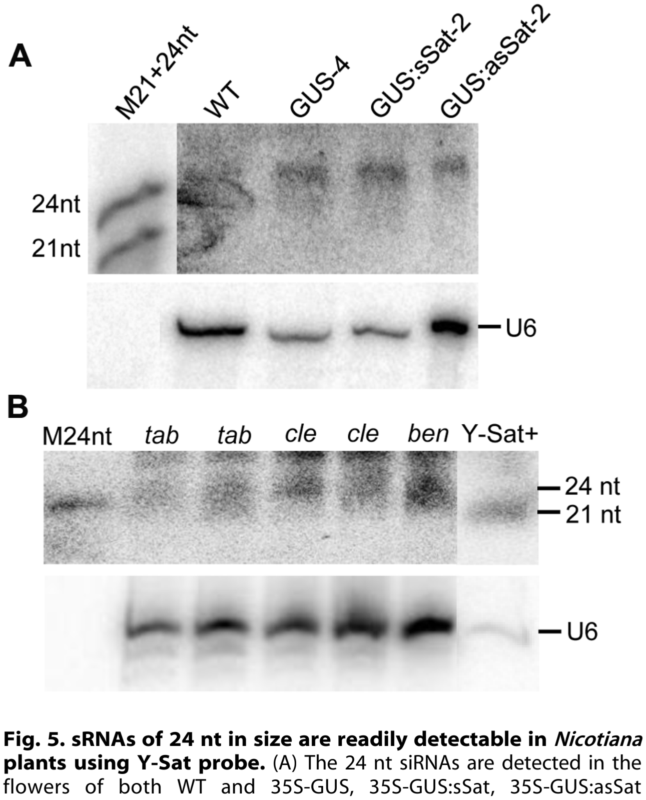

Fig. 5. sRNAs of 24 nt in size are readily detectable in *Nicotiana* plants using Y-Sat probe. (A) The 24 nt siRNAs are detected in the flowers of both WT and 35S-GUS, 35S-GUS:sSat, 35S-GUS:asSat transgenic *N. tabacum* plants. M21+24nt, 21 and 24-nt small RNA size marker. (B) 24 nt siRNAs are detectable in leaf tissues of three different *Nicotiana* species, *N. tabacum* (*tab*), *N. clevelandii* (*cle*), and *N. benthamiana* (*ben*). "Y-Sat+" is a RNA sample derived from a Y-Sat-infected *N. tabacum* plant, which was under-exposed to show the dominant ~21-nt Y-Sat-derived siRNAs. M24nt, 24-nt small RNA size marker. Both hybridized blots were treated with RNase A before exposure.
doi:10.1371/journal.pgen.1004906.g005

Host-derived Y-Sat-like 24 nt sRNAs are detected in *Nicotiana* plants by sRNA deep sequencing

Detection of 24-nt Y-Sat-like sRNAs in wild-type *Nicotiana* plants using northern blot hybridization prompted us to identify the nucleotide sequences of these sRNAs using Illumina sequencing. To avoid possible contamination by CMV Y-Sat (used in our Canberra laboratory), leaf samples from uninfected *N. tabacum* cv. Xanthi nc (*Nt-Xanthi*) grown under insect-proof conditions (in our Beijing laboratory where the CMV Shandong strain (SD-CMV) was used) were collected for sRNA extraction and sequencing. To investigate if CMV infection might affect the accumulation of Y-Sat-like sRNAs, we also sequenced sRNAs isolated from *Nt-Xanthi* infected with SD-CMVΔsatR, an infectious CMV clone devoid of SD-satRNA [29]. Approximately 17 and 23 million clean reads of sRNAs were obtained from the uninfected and SD-CMVΔsatR-infected plants, respectively, with 73.6% and 39% mapping perfectly to the uncompleted *N. tabacum* genome (ftp.sgn.cornell.edu) (Table 1). These *N. tabacum*-matching sRNAs were dominated by the 21 and 24-nt size classes (Table 1), consistent with the size distribution expected for plant sRNAs [30]. A large number of sRNAs matching the SD-CMV genome (27%) were identified in the sRNA sequencing data from SD-CMVΔsatR-infected plants, but none from the uninfected plants (Table 1). The majority (85.1%) of these SD-CMV-derived sRNAs was 21-22-nt in size, consistent with previous reports of sRNA distribution patterns from RNA viruses [19].

To identify Y-Sat-like sRNAs produced from the tobacco genome, the total sRNA reads were compared against the Y-Sat genome using BLASTN with varying statistical significance determined by E-values (i.e. the lower the E-value the greater the statistical significance of the match). Two additional CMV satRNA sequences (satCMV110 and SD-satRNA) were also used for comparison along with three randomly chosen similar sized (~360 nt) non-satRNA sequences (SD-CMV RNA1, *Influenza A virus* subtype H1N1 and *Rice stripe virus* RNA3 genome sequences) as negative controls.

The number of matching sRNAs was generally increased in the SD-CMVΔsatR-infected sample compared to the uninfected sample (Table 2 and Fig. 6). At a low-stringency E-value (le^{-2}), there was no significant difference in the number of sRNAs aligning to the three CMV satRNAs versus the three control sequences. However, using greater E-value stringencies (i.e. le^{-3}, le^{-4} and le^{-5}) to improve the alignment quality led to a higher frequency of sRNAs aligning to the Y-Sat genome than the control sequences in both the uninfected and particularly the SD-CMVΔsatRNA-infected samples (Table 2 and Fig. 6A, 6B). A large proportion of these Y-Sat-matching sRNAs were 24 nt in size (Table 2). The satCMV110 and SD-satRNA also had more

Table 1. Summary of sRNA sequencing data from uninfected and CMV△satRNA-infected *Nt. Xanthi*.

Sample	Total sRNA reads	Reads mapped to uncompleted *N. tabacum* genome	Reads mapped to SD-CMV genome
Uninfected *Nt-Xanthi*	17098983	12577289 (73.6%) (21 nt sRNA: 42.1%; 24 nt sRNA: 29.6%)	0
SD-CMV△satRNA-infected *Nt-Xanthi*	22654249	8845098 (39.0%)	6106661 (27.1%)

doi:10.1371/journal.pgen.1004906.t001

aligning sRNAs in the SD-CMV△satRNA-infected sample than the control sequences when using the higher alignment stringency (Table 2). In fact, no sRNA aligned to the relevant control sequences when using an E-value of $1e^{-5}$, with the exception of sRNAs aligning to the SD-CMV control sequence, which is a result of the SD-CMV infection (Table 2). The 14 unique Y-Sat-matching sRNA sequences aligned with both the 5′ and 3′ regions of the Y-Sat genome (Fig. 6C), suggesting the possible existence of long stretches of Y-Sat-like sequences in the *N. tabacum* genome. The five SD-satRNA-matching sRNAs identified in the SD-CMVΔsatR-infected sample ($1e^{-5}$) showed perfect sequence identity to the SD-satRNA genome, and had a size range of 23–26 nt (one 23-nt, one 26-nt and three 24-nt; Fig. 6B, the bottom panel; the colour-coded sequences). Three of these SD-matching sRNAs also aligned with the nt. 45–74 region of the Y-Sat and satCMV110 genomes (Fig. 6B, the top and middle panels; the colour-coded sequences). Taken together, the sRNA sequencing data indicated the presence of 24-nt sRNAs in the *N. tabacum* genome with sequence homologies to CMV satRNAs, particularly to Y-Sat, which could account for the 24-nt sRNA signals detected by northern blot (Fig. 5) and explain the DNA methylation of the Y-Sat sequence in the 35S-GUS:Sat transgenes (Fig. 3, 4 and S2 Fig.). It is noteworthy that the majority of the CMV satRNA-matching sRNAs could not be mapped to the uncompleted *N. tabacum* genome (S1 Table), indicating that these sRNAs are derived from the unannotated regions of the genome.

The CMV satRNA-matching sRNAs were more frequently aligning to certain regions of the satRNA genomes (Fig. 6 and S4 Fig.). Interestingly, these sRNA "hotspots" were relatively conserved among the three different satRNAs (boxed in Fig. 6 and S4 Fig.) and corresponded to conserved sequence regions among all CMV satRNA genomes (S5 Fig.). Among the three CMV satRNAs, SD-satRNA showed the most divergent distribution pattern of aligning sRNAs (Fig. 6A, B). Phylogenetic analysis of all published CMV satRNA sequences revealed that SD-satRNA, Y-Sat and satCMV110 are grouped into three distinct clusters, with SD-satRNA being the most ancient among the three (S6 Fig.).

The *Nicotiana* genome contains multiple Y-Sat-like DNA fragments

In plants, 24-nt siRNAs are usually derived from repetitive DNA regions such as transposable element (TE) sequences in the genome [30]. Multiple hybridizing DNA bands were detected in *N. tabacum*, *N. clevelandii*, and *N. benthamiana* plants by Southern blotting using the Y-Sat sequence as a probe (Fig. 7A). The different band patterns among the three *Nicotiana* species ruled out plasmid DNA contamination in the DNA samples. We also observed differences in the intensity of the hybridization signals, which suggested that Y-Sat-like DNA sequences exist in the *Nicotiana* species but with different copy numbers. Digestion of *N. tabacum* DNA with various restriction enzymes confirmed the existence of multiple copies of the Y-Sat-like DNA sequences (Fig. 7B).

Discussion

In this study we observed that a GUS transgene fused to the CMV Y-Sat sequence was transcriptionally repressed in *N. tabacum* plants in comparison to a non-fusion GUS transgene. Transcriptional gene silencing of a transgene is usually caused by promoter methylation and characterized by complete or near-complete silencing of the transgene in a subset of transgenic lines [31,32]. However, the 35S-GUS:Sat transgenes showed low but consistent levels of GUS expression across the independent transgenic lines. Furthermore, the 35S promoter was not methylated, suggesting that the repression of the 35S-GUS:Sat transgene is not due to conventional TGS.

Both McrBC PCR and bisulfite sequencing detected high levels of DNA methylation in the transcribed region of the 35S-GUS:Sat transgene, with the methylation restricted to the Y-Sat sequence. The extent of methylation in the Y-Sat sequence appeared to be

Table 2. Number of unique sRNA sequence hits to target sequence under different stringency of sequence match (E-value).

Matched sequence	sRNAs from uninfected *Nt-Xanthi*					sRNA from SD-CMV△satRNA-infected *Nt. Xanthi*			
	E-value	$1e^{-2}$	$1e^{-3}$	$1e^{-4}$	$1e^{-5}$	$1e^{-2}$	$1e^{-3}$	$1e^{-4}$	$1e^{-5}$
Y-Sat		170	148	40	0	1023	849	237	14
	% of 24nt sRNA	66%	70%	68%	0	52%	54%	54%	43%
satCMV110		169	19	1	1	1167	56	10	5
SD-satRNA		146	5	1	1	899	42	8	5
SD-CMV R1		149	14	0	0	12402	11156	10617	9673
H1N1		243	16	0	0	1459	110	10	0
RSVR3		205	33	2	0	1145	174	20	0

doi:10.1371/journal.pgen.1004906.t002

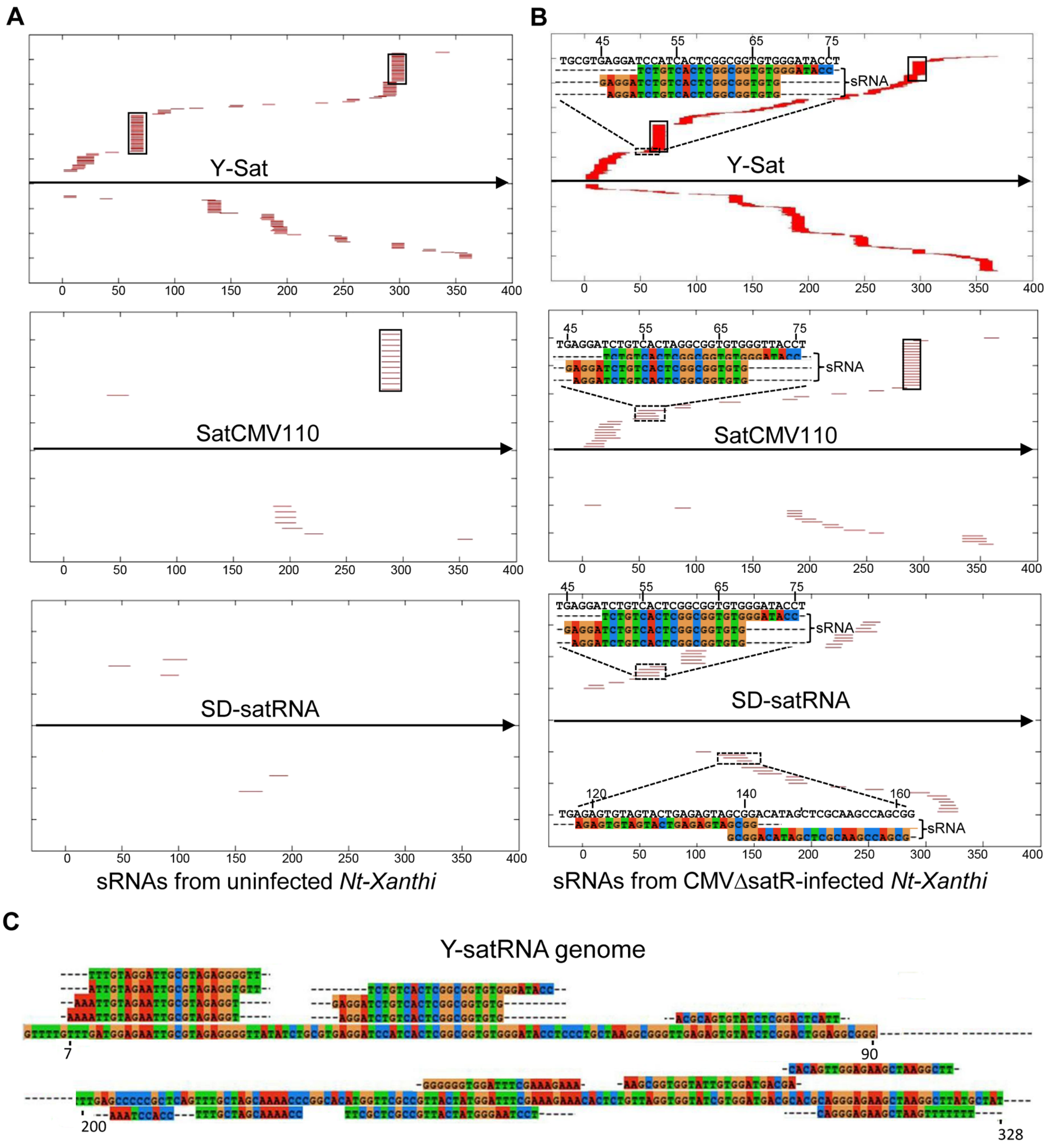

Fig. 6. Alignment of sRNAs to three CMV satRNA genome sequences at the E-value of 1e^{-3} (A and B) and 1e^{-5} (C). (A) sRNAs from uninfected *N. tabacum Xanthi* plants. (B) sRNAs from SD-CMV△satR-infected *N. tabacum Xanthi* plants. sRNAs matching the satRNA plus-strand and minus-strand are shown as short thin lines above and below the arrow-headed black lines, respectively. sRNAs mapping to the two most conserved regions of CMV satRNAs (nt. 60–80 and nt. 280–300; see S5 Fig.) are boxed. The five sRNAs perfectly matching to SD-satRNA, or nearly perfectly matching (for three of the five sRNAs) to Y-Sat and satCMV110, are shown as color-coded sequences and their respective nucleotide positions in the satRNA genome indicated. (C) Sequence and location of the 14 sRNAs identified from CMV△satR-infected *Nt-Xanthi* plants that map to the Y-Sat genome (the relevant part of the Y-Sat genome sequence, from nt. 1–90 and from nt. 200–328, is also shown).
doi:10.1371/journal.pgen.1004906.g006

inversely correlated to the level of 35S-GUS:Sat transgene expression, suggesting that this methylation may play a direct role in the repression of the transgene. How DNA methylation in the transcribed region represses the transcription of the 35S-GUS:Sat transgene remains to be investigated. However, our finding provides the first example of transgene repression associated with host-induced methylation targeted to a specific sequence in the transcribed region. The Y-Sat-specific DNA methylation is similar to that observed for the *Cereal yellow dwarf virus* (CYDV) satRNA sequence of a GUS:CYDV-satRNA fusion

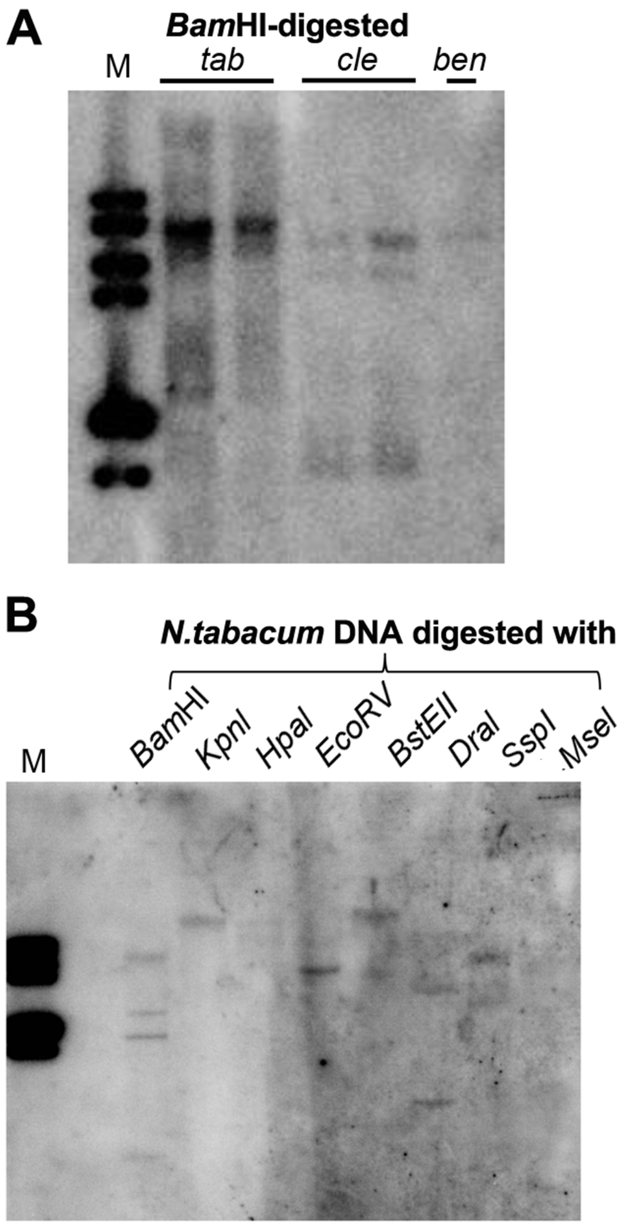

Fig. 7. Multiple DNA bands are detectable in *Nicotiana* species that show homology to Y-Sat. (A) One or multiple bands are detected in *Bam*HI-digested DNA of *N. tabacum* (*tab*), *N. clevelandii* (*cle*), and *N. benthamiana* (*ben*). (B) Digestion of *N. tabacum* DNA with different enzymes gives different band patterns. M, DNA size marker (1 kb DNA ladder).

doi:10.1371/journal.pgen.1004906.g007

transgene in *N. tabacum* [22]. The sequence-specific methylation of the CYDV sequence is caused by RdDM directed by sRNAs derived from replicating CYDV satRNA [22], also implicating RdDM as the cause of Y-Sat-specific methylation. However, methylation of the Y-Sat sequence occurred in the absence of replicating Y-Sat, suggesting that it was directed by host sRNA-induced RdDM.

Consistent with host-derived sRNA-induced RdDM being responsible for the methylation of the Y-Sat sequence, sRNAs with sequence homology to Y-Sat were readily detected by both northern blot hybridization and sRNA deep sequencing. Importantly, these sRNAs were primarily 24-nt in size, which are known to direct RdDM. This size distribution is different to that of sRNAs derived from replicating satRNAs, which are dominated by 21-22-nt size classes [19], ruling out contaminating satRNAs as the source of these sRNAs. In plants, 24-nt sRNAs are derived primarily from repetitive DNA sequences including TE sequences. Indeed, Southern blot analysis detected multiple DNA fragments in the *Nicotiana* genome with sequence homology to the Y-Sat sequence. Furthermore, the majority of the Y-Sat-matching sRNAs could not be mapped to the published *N. tabacum* genome sequence, suggesting that they are likely derived from highly repetitive regions of the genome that are usually difficult to assemble during genome sequencing and hence poorly annotated. Consistent with this possibility, a BLASTN search of the published genome sequences of *Nicotiana* species and two other Solanaceae species *Solanum lycopersicum* (tomato) and *S. tuberosum* (potato) did not yield any long (>20 bp) stretches of Y-Sat-like sequences, again implicating unassembled, repetitive regions as the source of the Y-Sat-matching sRNAs. Taken together, our results suggest that the *Nicotiana* genome contains Y-Sat-like DNA sequences, and that these sequences exist as repetitive DNA giving rise to 24-nt rasiRNA-like sRNAs that induce RdDM against the Y-Sat sequence in the 35S-GUS:Sat transgene. In addition to RdDM, 24-nt siRNAs have previously been shown to be capable of directing RNA degradation [33]. It would be interesting to examine if these host-derived 24-nt siRNAs play a role in the trilateral host-CMV-satRNA interaction by targeting satRNAs affecting satRNA accumulation.

Our results raise the possibility that CMV satRNAs originate from repetitive DNA elements in the *Nicotiana* genome. It has previously been speculated that satRNAs could be generated from the host plant under some unique conditions, such as helper virus infection [1]. Our sRNA sequencing showed that Y-Sat-like sRNAs accumulated at a much higher level in CMV-infected than uninfected *N. tabacum* plants. This implies that the Y-Sat-like repetitive DNA is transcriptionally repressed under normal conditions by DNA methylation, but is activated by CMV infection, possibly via the function of the 2b silencing suppressor that can repress DNA methylation in plants [34]. This would result in increased levels of transcript from repetitive DNA regions, including the Y-Sat-like DNA repeats, which could serve not only as substrate for sRNA production, but also as potential progenitor of CMV satRNAs. This scenario is consistent with the CMV-infected samples containing a much larger proportion of sRNAs that could not be mapped to the published *N. tabacum* genome than the uninfected samples (S1 Table). Viral replicases or RNA-dependent RNA polymerases have relatively high error rates [35], so replication of viral RNAs including satRNAs can be accompanied by a high rate of nucleotide mutations. Furthermore, for the host-derived progenitor RNAs to become satRNAs, they may need to undergo sequence changes to gain nucleotide motifs for efficient replication and encapsidation, and to gain stable secondary structures for resistance to nucleases. Thus, satRNAs, originating from the host, are likely to have substantial sequence variations from the original DNA or RNA sequences of the host genome. This would explain why most of the Y-Sat-matching *N. tabacum* sRNAs did not share perfect sequence identities with the Y-Sat genome. Our results do not rule out the possibility that the Y-Sat-like sequences in the *N. tabacum* genome was originally acquired from a viral genome like the non-retroviral RNA virus elements recently discovered in both plants and animals [36,37]. However, the fact that CMV satRNAs lack the ability to self-replicate and possess no sequence homology with their helper viruses, together with the previous observation of *de*

novo emergence of satRNAs during serial passaging plants with CMV infection under controlled environmental conditions [12], favours the view that the satRNAs originate from the sequence of the *Nicotiana* genome.

Our sRNA sequencing data indicated that different CMV satRNAs have different levels of sequence homology to the *N. tabacum* sRNAs. One possible explanation for this is that the different satRNAs originated from different *Nicotiana* species, or from different copies of repetitive DNA in one *Nicotiana* species, that have nucleotide variations. Another possibility is that these satRNAs originated from the *Nicotiana* genome at different times, with the more ancient ones having greater sequence divergence from the host genome than the more recent ones. This possibility is supported by the phylogenetic analysis of CMV satRNAs, showing the SD-satRNA to be more ancient than Y-Sat, which coincides with *N. tabacum* sRNAs matching better to Y-Sat than SD-satRNA.

In conclusion, by studying the abnormally repressed expression pattern of the 35S-GUS:Sat transgenes in *Nicotiana* plants, we have generated evidence indicating that CMV satRNAs have originated from repetitive DNA regions in the *Nicotiana* genome. Our study suggests that a small RNA sequence-based approach can be used to find the origin of satRNAs.

Materials and Methods

Plasmid construction, plant transformation, and *Agrobacterium* infiltration

The 35S-GUS-Ocs cassette, prepared using the pART7 plasmid [38], was previously described [39]. The cassette was excised using *Not*I and inserted into the *Not*I site of pART27 that contains a *NPTII* kanamycin resistance gene as the selectable marker for plant transformation [38], resulting in the 35S-GUS construct. For the 35S-GUS:sSat and 35S-GUS:asGUS constructs, the CMV 369-nt Y-Sat sequence was assembled using four long overlapping oligonucleotides (Y-Sat2, 3, 4 and 5; S2 Table), then PCR-amplified using Y-Sat1 and Y-Sat6 that contained a *Hin*dIII restriction site, and cloned into pGEMT-Easy vector (Promega). The full-length Y-Sat fragment was then excised with *Hin*dIII and inserted into the *Hin*dIII site between the GUS and Ocs sequence in the 35S-GUS-Ocs cassette in either the sense or antisense orientation. The resulting 35S-GUS:sSat-Ocs and 35S-GUS:as-Sat-Ocs cassettes were then excised with *Not*I and inserted into pART27 at the *Not*I site, forming the 35S-GUS:sSat and 35S-GUS:asSat constructs, respectively.

For transformation of *N. tabacum* Wisconsin 38 (W38), the constructs were introduced into *Agrobacterium tumefaciens* LBA4404 via triparental mating. For *Agrobacterium* infiltration assay, the constructs were transformed into *A. tumefaciens* GV3101.

Transformation of *N. tabacum* W38 was performed as previously described [10] using 50 mg/L kanamycin as the selective agent plus 150 mg/L timentin to inhibit *Agrobacterium* growth. Transformed plants with established roots were transferred to soil and grown at 25°C under natural light.

Agro-infiltration of *N. benthamiana* leaves was carried out essentially as described previously [40] with minor modifications. Basically, *A. tumefaciens* strains containing respective plant expression constructs, including a green florescent protein (GFP) construct as a visual marker and a P19 construct as the RNA silencing suppressor [41], were grown overnight at 28°C in Luria-Bertani medium (LB) containing appropriate antibiotics. After centrifugation, *Agrobacterium* cells were re-suspended in buffer containing 10 mM $MgCl_2$ and 150 μM acetosyringone, to a final optical density at 600 nm (OD_{600}) of 1.0. *Agrobacterium* cell suspension containing either the 35S-GUS or the 35S-GUS:sSat construct was mixed with the GFP and P19 cell suspensions at a 1:1:1 ratio, incubated at room temperature for ~3 h, and then infiltrated into expanded *N. benthamiana leaves* using a flat-pointed syringe. For protein and RNA isolation, agro-infiltrated leaf sections at 4 or 5 days post agroinfiltration (dpa) were visualized under a blue light torch (NightSea^{TM}, DFP-1^{TM}, for exciting green light emission), excised with a pair of scissors and immediately frozen in liquid nitrogen.

Southern and Northern blot hybridization

Genomic DNA used for Southern blot hybridization and bisulfite conversion was isolated using the CTAB method described by Draper and Scott [42]. Restriction digestion of DNA (~20 μg), purification, agarose gel electrophoresis and Southern blot hybridization were essentially as previously described [39]. A *Hin*dIII fragment containing the full-length Y-Sat sequence was excised from the pGEM-T Easy clone described above, and used for preparing a ^{32}P-labelled probe using the Megaprime DNA Labelling System (Amersham Biosciences). Hybridized membranes were washed with 1×SSC first at room temperature for 20 min, then at 50°C for 20 min, and finally at 60–65°C for 30 min. The blots were visualized using a phosphor-imager (FLA-5000, Fuji Photo Film).

RNA used for small RNA northern blot hybridization was isolated from agro-infiltrated *N. benthamiana* leaf tissues or transgenic *N. tabacum* leaves using Trizol Reagent (Invitrogen). For northern blot detection of small RNAs from *N. tabacum* flowers, total RNAs was isolated using Trizol reagent, high-molecular-weight RNA removed by precipitation with 5% polyethylenglycol 8000 (PEG 8000) and 0.5 M NaCl on ice for 20 min, and small RNAs recovered from the supernatant by precipitation with 3 volumes of ethanol at −20°C for 1 hr. RNA used for northern blot analysis of GUS or GUS:Sat fusion mRNA was isolated using the hot-phenol extraction method [43]. sRNA northern blot hybridization was performed as described using ^{32}P-labelled in-vitro antisense transcript of full-length Y-Sat or GUS coding sequence that were fragmented with Na_2CO3 treatment [10]. mRNA northern blot hybridization was carried out as previously described [39] using the same full-length antisense GUS RNA (unfragmented) as probe.

MUG assay

GUS activities were determined using the kinetic MUG (4-methylumbelliferyl-β- glucuronide) assay according to Chen et al. [39]. GUS activities for Fig. 1B was calculated according to Chen at al. [39], with the Y-axis as pmol MU per min per 5 μg protein. GUS activities for the remaining figures were presented as slope value per 5 μg protein [44].

Nuclear run-on assay

Nuclei were isolated from approximately 4 g of fresh *N. tabacum* leaves and used for nuclear run-on assay according to the procedure described in Meng and Lemaux [45]. The full-length GUS gene, the elongation factor 1α (EF1α) gene (used as internal reference), and the *Fusarium oxysporum* FKS1 gene (used as negative control) sequences, were amplified by PCR using the following primers (primer sequences are shown in S2 Table): M13-F and M13-R (from a pGEM-GUS plasmid), EF1α-F and EF1α-R, and FKS1-F and FKS1-R, respectively. The PCR product was separated in 1% agarose gel, denatured, neutralized and then blotted onto Hybond N$^+$ membrane using 20× SSC following the manufacturer's instruction. The membrane was hybridized with

the nuclear run-on transcript according to Meng and Lemaux [45] and the hybridizing signals were visualized using a phosphor-imager (FLA-5000, Fuji Photo Film).

McrBC digestion PCR

Genomic DNA (~2 μg) from transgenic 35S-GUS:sSat *N. tabacum* plants, was mixed with 1×NEB buffer 2, 0.1 μg/μl BSA, 1 mM GTP in 94 μl volume, which was divided in two equal 47 μl aliquots. To one aliquot 3 μl McrBC enzyme (NEB, 10 units/μl) was added, to the other 3 μl of H_2O. Both were incubated at 37°C overnight and then diluted with H_2O to 100 μl. Four μl was used for each PCR reaction, which was performed using the following cycles: 1 cycle of 95°C for 3 min, 33 cycles of 95°C for 30 sec, 56°C for 45 sec, and 72°C for 90 sec, followed by one cycle of 72°C for 10 min. Sequences of the McrBC PCR primers are listed in S2 Table. Real-time PCR was performed using the Rotor-Gene 6000 (Corbett Life Science, San Francisco, USA) real-time rotary analyser using SYBR Green reagent and Platinum Taq polymerase (Invitrogen) in four technical replicates for each sample.

Bisufite sequencing

Bisulfite conversion of transgenic *N. tabacum* genomic DNA (~4 μg) was performed as previously described [33]. The bisulfite-treated DNA was purified using Qiagen PCR Purification kit. Primer design (sequences shown in S2 Table), nested PCR and direct sequencing of PCR products were as previously described [44].

Small RNA sequencing and data analysis

Total RNA was isolated from wild-type *Nt-Xanthi* plants and SD-CMVΔSat-infected plants using hot-phenol extraction and small RNAs were prepared as described previously [46]. Small RNA library construction and Illumina sequencing was performed by BGI (http://www.genomics.cn/en/index). For data analysis, adapter and low-quality sequences were removed and cleaned reads used for length distribution and subsequent analysis. Overlapping sequences between different libraries were identified using Perl script (http://www.perl.org/). All clean reads were mapped to the uncompleted *Nt-Xanthi* genome (ftp.sgn.cornell.edu) using SOAP (http://soap.genomics.org.cn/), and all perfectly mapped sequences were used to determine the mapping ratio in different samples.

All clean reads matching to satRNA and control sequences was examined using BLASTN (http://blast.ncbi.nlm.nih.gov/Blast.cgi) with different E-values ($1e^{-2}$, $1e^{-3}$, $1e^{-4}$ and $1e^{-5}$) to determine different degrees of similarity. ClustalX (http://www.clustal.org/) was used for sequence alignments and phylogenetic analysis of satRNA genomes.

Supporting Information

S1 Fig The difference in GUS expression between 35S-GUS and 35S-GUS:sSat transgenes is diminished in *Agrobacterium*-infiltrated *N. benthamiana* leaf tissues. (A) MUG assay of leaf tissues agro-infiltrated with 35S-GUS + P19 (a) or 35S-GUS:sSat + P19 (b). (B) Northern blot hybridization using antisense GUS RNA as probe. (C) Detection of small RNAs in agro-infiltrated tissues at 5 dpa (days post agro-infiltration) using northern blot hybridization. The numbers (1, 2, 3, 4, 5 and 6) denote a specific leaf of which one half was infiltrated with 35S-GUS + P19 (a) and the other half with 35S-GUS:sSat + P19 (b). Transient expression of agro-infiltrated transgenes is not expected to be affected by TGS, and PTGS (or sense cosuppression) is minimized by co-infiltrating the construct expressing P19, a strong viral suppressor of RNA silencing. Note that the difference in GUS expression between the two transgenes in this transient assay is much reduced in comparison to that in stably transformed plants (Fig. 1). The slightly lower expression level of the 35S-GUS:sSat transgene could be due to the higher level of 21-nt sRNAs shown in (C), as 21-nt sRNA-directed PTGS of the agro-infiltrated transgenes was unlikely to be completely prevented by P19.
(TIF)

S2 Fig Methylation analysis of the 35S-GUS:asSat transgene in transgenic *N. tabacum*. (A) McrBC PCR of the 35S promoter, GUS coding and Y-Sat sequences. (B) MUG assay of GUS activity in the transgenic plants used for the McrBC PCR analysis. Note that there is no clear difference in the PCR band intensity between McrBC-digested (+) and undigested (−) samples for the 35S and GUS sequences, but there is a clear reduction in amplification for the Y-Sat sequence upon McrBC digestion. Also note that the 35S-GUS:asSat-1a plant appears to show the strongest methylation in the Y-Sat sequence, and this coincides with the lowest level of GUS expression among the three 35S-GUS:asSat plants.
(TIF)

S3 Fig Bisulfite sequencing shows no significant cytosine methylation in the 35S promoter and the GUS coding regions of the 35S-GUS:sSat transgene. (A) The three bisulfite-sequenced regions in the 35S-GUS:sSat transgene, I, II, and III, are indicated. (**B**) Sequencing trace file of bisulfite PCR product from region I (35S promoter). (C) Sequencing trace file of bisulfite PCR product from region II (35S-GUS junction). The numbers in (B) and (C) indicate nucleotide positions of the bisulfite sequenced regions in the 35S promoter sequence. The GUS sequence in the 35S-GUS junction region is boxed (C). Cytosine residues of the original sequences are in bold-blue C letter.
(TIF)

S4 Fig Alignment of sRNAs from uninfected *N. tabacum Xanthi* to satCMV110 and SD-satRNA genome sequences at the E-value of $1e^{-2}$. sRNAs matching the plus strand satRNA sequence are shown as short thin lines above the arrow-headed black lines, and those matching the minus-strand satRNA sequence shown below the arrow-headed lines. sRNAs mapped to the two most conserved regions of CMV satRNAs (positions around nt. 60–80 and nt. 280–300) are boxed.
(TIF)

S5 Fig Nucleotide sequence alignment of all CMV satRNA genome sequences listed in the subviral RNA database at http://subviral.med.uottawa.ca/cgi-bin/home.cgi?oldURL=1. Sequences of SD-satRNA, Y-Sat and satCMV110 are highlighted in blue. Conserved nucleotides among all satRNAs are indicated with asterisks at the bottom. Two red boxes mark the regions corresponding to the most conserved sRNA hotspots in Fig. 6 and S4 Fig.
(TIF)

S6 Fig Phylogenetic relationship of all CMV satRNA genomes from subviral RNA database at http://subviral.med.uottawa.ca/cgi-bin/home.cgi?oldURL=1. Accession numbers of satRNA strains are shown. SD-satRNA, Y-Sat and satCMV110 are indicated.
(TIF)

S1 Table A large proportion of the unique CMV satRNA-matching sRNA sequences cannot be mapped to the uncompleted *N. tabacum* genome.
(DOCX)

S2 Table Oligonucleotide sequences.
(DOCX)

Acknowledgments

We would like to thank Chikara Masuta for providing the Y-Sat infectious clone. We also thank Wanxia Shen for assistance with small RNA northern blot hybridization, Claire Anderson with PCR analysis, and three reviewers for constructive comments.

Author Contributions

Conceived and designed the experiments: MBW HSG RZ ESD. Performed the experiments: KZ JHZ NAS US YYF MBW. Analyzed the data: KZ JHZ NAS US YYF MBW HSG. Contributed reagents/materials/analysis tools: MBW HSG. Wrote the paper: MBW HSG ESD RZ JHZ.

References

1. Simon AE, Roossinck MJ, Havelda Z (2004) Plant virus satellite and defective interfering RNAs: new paradigms for a new century. Annu Rev Phytopathol 42: 415–437.
2. Hu C-C, Hsu Y-H, Lin N-S (2009) Satellite RNAs and satellite viruses of plants. Viruses 1: 1325–1350.
3. Roossinck M, Sleat D, Palukaitis P (1992) Satellite RNAs of plant viruses: structures and biological effects. Microbiological Reviews 56: 265–279.
4. Garcia-Arenal F, Palukaitis P (1999) Structure and functional relationships of satellite RNAs of cucumber mosaic virus. Satellites and Defective Viral RNAs: Springer. pp. 37–63.
5. Wang X-B, Wu Q, Ito T, Cillo F, Li WX, et al. (2010) RNAi-mediated viral immunity requires amplification of virus-derived siRNAs in Arabidopsis thaliana. Proc Natl Acad Sci USA 107: 484–489.
6. Choi SH, Seo JK, Kwon SJ, Rao AL (2012) Helper virus-independent transcription and multimerization of a satellite RNA associated with cucumber mosaic virus. J Virol 86: 4823–4832.
7. Chaturvedi S, Kalantidis K, Rao AL (2014) A bromodomain-containing host protein mediates the nuclear importation of a satellite RNA of Cucumber mosaic virus. J Virol 88: 1890–1896.
8. Collmer CW, Howell SH (1992) Role of satellite RNA in the expression of symptoms caused by plant viruses. AnnRev Phytopathol 30: 419–442.
9. Shimura H, Pantaleo V, Ishihara T, Myojo N, Inaba J-i, et al. (2011) A viral satellite RNA induces yellow symptoms on tobacco by targeting a gene involved in chlorophyll biosynthesis using the RNA silencing machinery. PLoS Pathog 7: e1002021.
10. Smith NA, Eamens AL, Wang M-B (2011) Viral small interfering RNAs target host genes to mediate disease symptoms in plants. PLoS Pathog 7: e1002022.
11. Simon AE, Howell SH (1986) The virulent satellite RNA of turnip crinkle virus has a major domain homologous to the 3'end of the helper virus genome. The EMBO J 5: 3423.
12. Hajimorad M, Ghabrial S, Roossinck M (2009) De novo emergence of a novel satellite RNA of cucumber mosaic virus following serial passages of the virus derived from RNA transcripts. Arch Virol 154: 137–140.
13. Collmer CW, Hadidi A, Kaper J (1985) Nucleotide sequence of the satellite of peanut stunt virus reveals structural homologies with viroids and certain nuclear and mitochondrial introns. Proc Natl Acad Sci USA 82: 3110–3114.
14. Meister G, Tuschl T (2004) Mechanisms of gene silencing by double-stranded RNA. Nature 431: 343–349.
15. Baulcombe D (2005) RNA silencing. Trends Biochem Sci 30: 290–293.
16. Baulcombe D (2004) RNA silencing in plants. Nature 431: 356–363.
17. Eamens A, Wang M-B, Smith NA, Waterhouse PM (2008) RNA silencing in plants: yesterday, today, and tomorrow. Plant Physiol 147: 456–468.
18. Bartel DP (2004) MicroRNAs: genomics, biogenesis, mechanism, and function. Cell 116: 281–297.
19. Ding S-W, Voinnet O (2007) Antiviral immunity directed by small RNAs. Cell 130: 413–426.
20. Matzke MA, Birchler JA (2005) RNAi-mediated pathways in the nucleus. Nat Rev Genet 6: 24–35.
21. Zhang H, Zhu J-K (2011) RNA-directed DNA methylation. Curr Opin Plant Biol 14: 142–147.
22. Wang M-B, Wesley SV, Finnegan EJ, Smith NA, Waterhouse PM (2001) Replicating satellite RNA induces sequence-specific DNA methylation and truncated transcripts in plants. RNA 7: 16–28.
23. Masuta C, Kuwata S, Takanami Y (1988) Effects of extra 5'non-viral bases on the infectivity of transcripts from a cDNA clone of satellite RNA (strain Y) of cucumber mosaic virus. J Biochem 104: 841–846.
24. Park YD, Papp I, Moscone E, Iglesias V, Vaucheret H, et al. (1996) Gene silencing mediated by promoter homology occurs at the level of transcription and results in meiotically heritable alterations in methylation and gene activity. Plant J 9: 183–194.
25. Clark SJ, Harrison J, Paul CL, Frommer M (1994) High sensitivity mapping of methylated cytosines. Nucleic Acids Res 22: 2990–2997.
26. Le TN, Schumann U, Smith NA, Tiwari S, Au P, et al. (2014) DNA demethylases target promoter transposable elements to positively regulate stress responsive genes in Arabidopsis. Genome Biol 15: 458.
27. Mosher RA, Melnyk CW, Kelly KA, Dunn RM, Studholme DJ, et al. (2009) Uniparental expression of PolIV-dependent siRNAs in developing endosperm of Arabidopsis. Nature 460: 283–286.
28. Wang H, Zhang X, Liu J, Kiba T, Woo J, et al. (2011) Deep sequencing of small RNAs specifically associated with Arabidopsis AGO1 and AGO4 uncovers new AGO functions. Plant J 67: 292–304.
29. Hou WN, Duan CG, Fang RX, Zhou XY, Guo HS (2011) Satellite RNA reduces expression of the 2b suppressor protein resulting in the attenuation of symptoms caused by Cucumber mosaic virus infection. Mol Plant Pathol 12: 595–605.
30. Kasschau KD, Fahlgren N, Chapman EJ, Sullivan CM, Cumbie JS, et al. (2007) Genome-wide profiling and analysis of Arabidopsis siRNAs. PLoS Biol 5: e57.
31. Waterhouse PM, Wang M-B, Lough T (2001) Gene silencing as an adaptive defence against viruses. Nature 411: 834–842.
32. Matzke MA, Aufsatz W, Kanno T, Mette MF, Matzke AJ (2002) Homology-dependent gene silencing and host defense in plants. Advances in Genetics 46: 235–275.
33. Wang M-B, Helliwell CA, Wu L-M, Waterhouse PM, Peacock WJ, et al. (2008) Hairpin RNAs derived from RNA polymerase II and polymerase III promoter-directed transgenes are processed differently in plants. RNA 14: 903–913.
34. Duan C-G, Fang Y-Y, Zhou B-J, Zhao J-H, Hou W-N, et al. (2012) Suppression of Arabidopsis ARGONAUTE1-mediated slicing, transgene-induced RNA silencing, and DNA methylation by distinct domains of the Cucumber mosaic virus 2b protein. Plant Cell 24: 259–274.
35. Steinhauer D, Holland J (1987) Rapid evolution of RNA viruses. Ann Rev Microbiol 41: 409–431.
36. Horie M, Honda T, Suzuki Y, Kobayashi Y, Daito T, et al. (2010) Endogenous non-retroviral RNA virus elements in mammalian genomes. Nature 463: 84–87.
37. Chiba S, Kondo H, Tani A, Saisho D, Sakamoto W, et al. (2011) Widespread endogenization of genome sequences of non-retroviral RNA viruses into plant genomes. PLoS Pathog 7: e1002146.
38. Gleave AP (1992) A versatile binary vector system with a T-DNA organisational structure conducive to efficient integration of cloned DNA into the plant genome. Plant Mol Biol 20: 1203–1207.
39. Chen S, Helliwell CA, Wu L-M, Dennis ES, Upadhyaya NM, et al. (2005) A novel T-DNA vector design for selection of transgenic lines with simple transgene integration and stable transgene expression. Funct Plant Biol 32: 671–681.
40. Cazzonelli CI, Velten J (2006) An in vivo, luciferase-based, Agrobacterium-infiltration assay system: implications for post-transcriptional gene silencing. Planta 224: 582–597.
41. Wood CC, Petrie JR, Shrestha P, Mansour MP, Nichols PD, et al. (2009) A leaf-based assay using interchangeable design principles to rapidly assemble multistep recombinant pathways. Plant Biotech J 7: 914–924.
42. Draper J, Scott R, Armitage P, Walden R (1988) Plant genetic transformation and gene expression: a laboratory manual: Blackwell Scientific Publications.
43. De Vries S, Hoge H, Bisseling T (1989) Isolation of total and polysomal RNA from plant tissues. Plant Molecular Biology Manual: Springer. pp. 323–335.
44. Finn TE, Wang L, Smolilo D, Smith NA, White R, et al. (2011) Transgene expression and transgene-induced silencing in diploid and autotetraploid Arabidopsis. Genetics 187: 409–423.
45. Meng L, Lemaux P (2003) A simple and rapid method for nuclear run-on transcription assays in plants. Plant Mol Biol Rep 21: 65–71.
46. He X-F, Fang Y-Y, Feng L, Guo H-S (2008) Characterization of conserved and novel microRNAs and their targets, including a TuMV-induced TIR–NBS–LRR class R gene-derived novel miRNA in Brassica. FEBS letters 582: 2445-2452.
